# Development and Validation of a 7-Gene Inflammatory Signature Forecasts Prognosis and Diverse Immune Landscape in Lung Adenocarcinoma

**DOI:** 10.3389/fmolb.2022.822739

**Published:** 2022-03-15

**Authors:** Aitao Nai, Feng Ma, Zirui He, Shuwen Zeng, Shoaib Bashir, Jian Song, Meng Xu

**Affiliations:** ^1^ Department of Oncology, The First Affiliated Hospital of Jinan University, Guangzhou, China; ^2^ Department of Oncology, ZhongShan Torch Development Zone Hospital, Zhongshan, China

**Keywords:** lung adenocarcinoma, inflammatory response, multi-gene signature, prognostic biomarker, immune status, chemosensitivity

## Abstract

**Background:** Inflammatory responses are strongly linked with tumorigenesis and cancer development. This research aimed to construct and validate a novel inflammation response–related risk predictive signature for forecasting the prognosis of patients with LUAD.

**Methods:** Differential expression analysis, univariate Cox, LASSO, and multivariate Cox regression analyses of 200 inflammatory response–related genes (IRRG) were performed to establish a risk predictive model in the TCGA training cohort. The performance of the IRRG model was verified in eight GEO datasets. GSEA analysis, ESTIMATE algorithms, and ssGSEA analysis were applied to elucidate the possible mechanisms. Furthermore, the relationship analysis between risk score, model genes, and chemosensitivity was performed. Last, we verified the protein expression of seven model genes by immunohistochemical staining or Western blotting.

**Results:** We constructed a novel inflammatory response–related 7-gene signature (MMP14, BTG2, LAMP3, CCL20, TLR2, IL7R, and PCDH7). Patients in the high-risk group presented markedly decreased survival time in the TCGA cohort and eight GEO cohorts than the low-risk group. Interestingly, multiple pathways related to immune response were suppressed in high-risk groups. The low infiltration levels of B cell, dendritic cell, natural killer cell, and eosinophil can significantly affect the unsatisfactory prognosis of the high-risk group in LUAD. Moreover, the tumor cells’ sensitivity to anticancer drugs was markedly related to risk scores and model genes. The protein expression of seven model genes was consistent with the mRNA expression.

**Conclusion:** Our IRRG prognostic model can effectively forecast LUAD prognosis and is tightly related to immune infiltration.

## Introduction

Lung cancer is a clinical malignancy with the third highest morbidity and the highest mortality worldwide (Siegel et al., 2021). Histologically, lung adenocarcinoma (LUAD) occupies about half of all lung carcinoma (Lou et al., 2021). Despite considerable advances in targeted therapy and immunotherapy, the overall 5-year survival rate for LUAD is about 15% (Chen et al., 2019). Recently, with the progress of RNA sequencing technology, an increasing number of key genes have been discovered, and their abnormal expression can drive cancer initiation and development and predict the patient’s prognosis. However, as we all know, tumors are highly polygenic, and a single gene cannot well forecast the prognosis of tumor patients (Zhang et al., 2020). Hence, there is an urgent need for a more effective prognostic model integrated with multiple genes for prognosis prediction and potential therapeutic targets in patients with LUAD.

Inflammation is a beneficial response of the body to injury and a vital segment of the immunity response (Hagemann et al., 2007). The role of inflammation in tumorigenesis and cancer development has been the focal point of considerable research (Greten and Grivennikov, 2019). The cancer-promoting and cancer-suppressive roles of inflammation in malignancies have been proposed (Lin et al., 2021; Haabeth et al., 2016). On the one hand, inflammation promotes cancer cell proliferation and metastasis (Zhao et al., 2020). On the other hand, pro-inflammatory cytokines, such as TNFα, IL-6, IL-1a, and IL-1β, can promote anticancer immunity (Haabeth et al., 2012). Recent studies demonstrated that some prognostic models based on IRRG could accurately forecast the prognostic of liver or colon cancer (Liang et al., 2021; Lin et al., 2021). Nevertheless, the expression and prognostic value of IRRG in LUAD remain obscure.

Here, we established a novel inflammation response-related risk predictive signature for forecasting the prognosis of patients with LUAD, and the model’s performance was verified in multiple GEO cohorts. Besides, we explored the relevance between the risk score of prognostic models and immune infiltration to illustrate the possible mechanisms of the discrepancy in survival outcome between the high- and low-risk groups. Finally, we evaluated the relationship between risk score, model genes, and chemosensitivity. Collectively, our study suggests that the prognostic model can provide a novel and valuable reference for prognosis judgment in LUAD.

## Materials and Methods

### Data Acquisition

The clinical and RNA-seq data of LUAD in the TCGA dataset were acquired from the UCSC Xena website (http://xena.ucsc.edu/). Data are presented as Log2(FPKM+1). Microarray data of LUAD were downloaded from the GEO dataset (https://www.ncbi.nlm.nih.gov/geo/). 200 IRRG were downloaded from the MSigDB platform (http://www.gsea-msigdb.org/gsea/index.jsp) (Supplementary Table S1). The data of tumor cells’ sensitivity to chemotherapy drugs were downloaded from the CellMiner database (https://discover.nci.nih.gov/cellminer/).

### GSEA

We carried out the GSEA analysis between normal and LUAD tissues in the TCGA dataset and five GEO datasets (GSE75037, GSE63459, GSE43458, GSE31210, and GSE30219) according to Hallmark gene set (h.all.v7.4, http://www.gsea-msigdb.org/gsea/index.jsp). In the TCGA cohort, after excluding duplicate patients, the GSEA analysis of transcriptomic data of 59 normal and 513 LUAD samples was conducted. Table 1 shows the number of samples included in this study for all datasets. Adjusted *p*-value < 0.05 was identified as statistically different.

**TABLE 1:** Number of samples incorporated in this study.

Data set	Number of normal samples	Number of LUAD samples	Number of survival analysis
TCGA	59	513	500
GSE75037	83	83	—
GSE63459	32	33	—
GSE43458	30	80	—
GSE31210	20	226	226
GSE30219	14	85	85
GSE72094	—	—	398
GSE68465	—	—	442
GSE41271	—	—	182
GSE42127	—	—	133
GSE50081	—	—	127
GSE26939	—	—	115

### Establishment of the IRRG Prognostic Signature

To begin with, differential expression analysis and survival analysis of 200 IRRG in the TCGA cohort were executed. |logFC| > 0.5 and adjusted *p*-value < 0.05 were considered as differentially expressed genes (Li et al., 2021). Genes whose *p*-values for both univariate Cox regression analyses and Kaplan–Meier (KM) survival analysis were less than 0.05 were identified as prognostic genes. Then, after LASSO and multivariate Cox regression analyses, seven genes were applied to construct an IRRG prognostic model in the TCGA cohort. The risk score calculation formula is as follows: Risk score = (0.15149541 × expression of MMP14)—(0.13431568 ×expression of BTG2) + (0.10373004 × expression of LAMP3) + (0.13130051 × expression of CCL20)—(0.20082994 × expression of TLR2)—(0.20672746 × expression of IL7R) + (0.24153768 × expression of PCDH7).

### Assessment and Validation of the IRRG Model

All samples were distinguished into high-risk and low-risk groups according to the best cut-off value (1.04349) in the training cohort. Next, we performed survival analysis to assess the prognostic significance among two groups through “survminer” and “survival” R packages in the TCGA training cohort and eight validation cohorts (GSE30219, GSE31210, GSE72094, GSE68465, GSE41271, GSE42127, GSE50081, and GSE26939) (Wu et al., 2021). ROC analysis was utilized to estimate the forecasting capability of the IRRG model. Univariate and multivariate Cox analyses were used to investigate whether the survival prognosis of the risk score was independent of other clinicopathological characteristics. Finally, we carried out a meta-analysis through “meta” packages in R software (Peng et al., 2021). The random effects model was adopted according to *I* ^*2*^ > 50% and *p* < 0.05 (Wang Y. C et al., 2019).

### Nomogram Analysis

A prognostic nomogram model integrated with a risk score, age, gender, stage, T classification, N classification, and M classification was established to more accurately forecast the prognosis of LUAD patients from the TCGA cohort using “regplot” R packages. “rms” R package was utilized to plot the calibration curve. “survivalROC” R package was used to assess the forecasting capability of the risk score among diverse clinicopathological features.

### Immune Feature Analysis

First, GSEA was executed to elucidate the enriched pathways between high- and low-risk groups according to KEGG (c2.cp.kegg.v7.4) and GO:BP (c5.go.bp.v7.4) gene set. Then, we compared the immune score, stromal score, and ESTIMATE score between high- and low-risk groups through the “Estimate” R package. Subsequently, single sample gene set enrichment analysis (ssGSEA) was executed using the “GSVA” R package to unambiguously present the infiltrating score of 28 tumor-infiltrating immune cells between high- and low-risk groups (Zhao et al., 2021). Finally, we investigated the prognostic value of 28 infiltrating immune cells and their correlation with the risk score using the Spearman method.

### The Relationship Between Risk Score, Model Genes, and Chemosensitivity

The NCI-60 was used to evaluate the relationship between risk score, model genes, and chemosensitivity (Nai et al., 2021). After removing tumor cell lines with more than 80% data loss, a total of 59 tumor cell lines and 792 chemotherapeutic or targeted agents were included in the analysis (Supplementary Table S2). The correlation analysis was performed utilizing the Spearman method.

### The Expression Validation of Model Genes

We verified the protein expression of MMP14, PCDH7, and LAMP3 using immunohistochemical staining images from the HPA database (https://www.proteinatlas.org/). Moreover, we also verified the differential expression of CCL20, BTG2, IL7R, and TLR2 between normal bronchial epithelial cell line (16-HBS) and lung adenocarcinoma cell line (A549) by utilizing Western blotting. 16-HBS and A549 cell lines were purchased from the Cell Bank of the Type Culture Collection of the Chinese Academy of Sciences (Shanghai, China). Protein concentrations were evaluated through the BCA Assay kit (CWBIO). Western blotting was performed as seen previously (Wang et al., 2020). Briefly, 15 ug proteins were loaded onto 8–15% polyacrylamide gels. After transfer, the NC membranes (Boster) were blocked with 5% skimmed milk at room temperature for 2 h. Next, membranes were incubated with primary antibodies [CCL20 (Affinity, 1:300), BTG2 (proteintech, 1:300), IL7R (Affinity, 1:300), TLR2 (proteintech, 1:1000)], and GAPDH (Elabscience, 1:3000) for 2 h at room temperature and then at 4°C overnight. Subsequently, the membranes were incubated with horseradish peroxidase-conjugated goat anti-rabbit (CWBIO, 1:1000) at room temperature for 2 h. Finally, the bands were exposed with ECL reagents (CWBIO). ImageJ software was applied to perform the gray value analysis.

### Statistical Analysis

We carried out statistical analyses using R software (version 4.1.1). All R codes are presented in Supplementary Table S3. Risk score differences in different clinicopathological characteristics and diverse infiltrating immune cells were examined by using an unpaired *t*-test. Log-rank test was utilized to elucidate the survival differences. Moreover, the statistical differences of Western blotting results were evaluated using the Student’s *t*-test through GraphPad Prism 9.3 Software. *p* less than 0.05 was regarded as statistically different.

## Results

### Inflammatory Response Pathway Is Suppressed in LUAD

**FIGURE 1:**
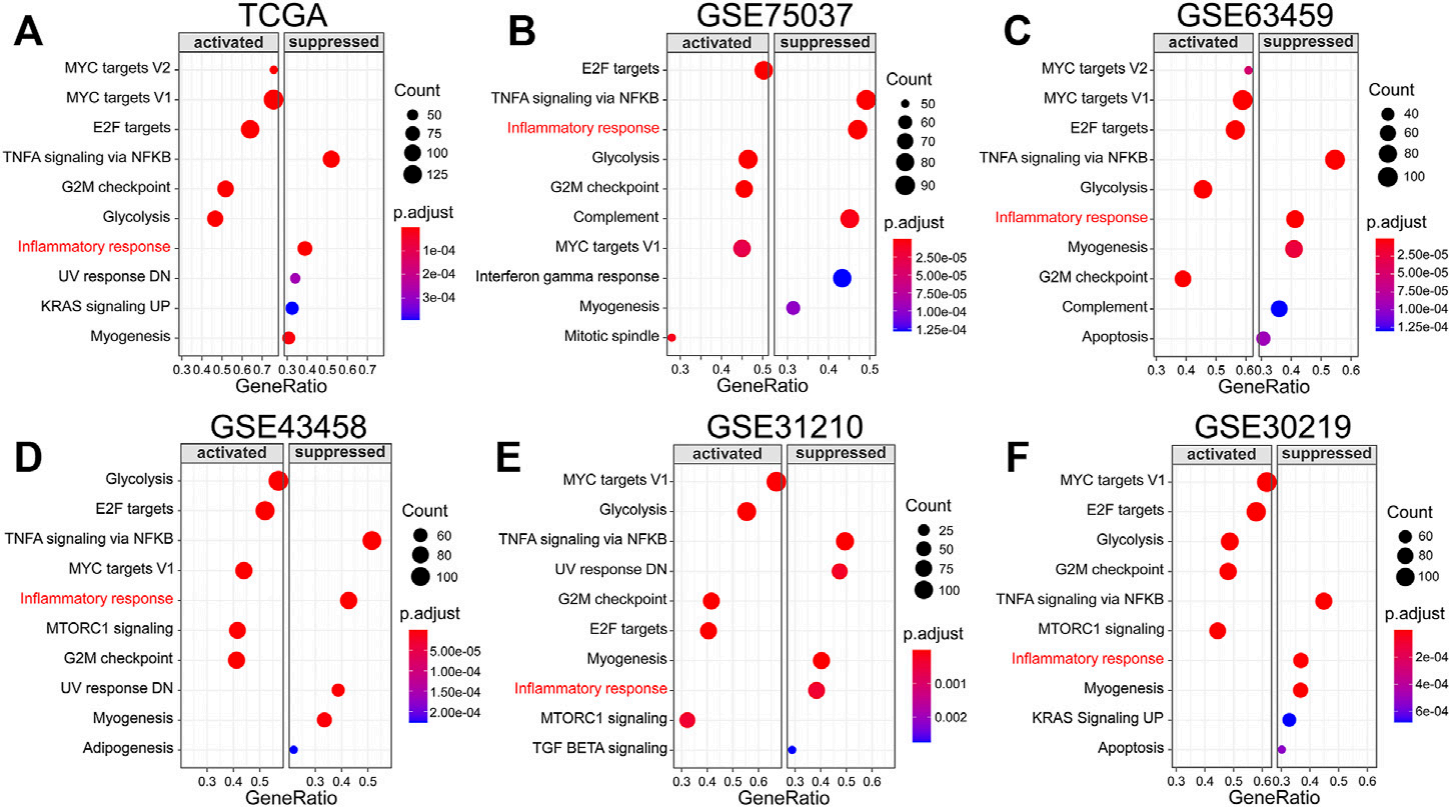
Inflammatory response pathway is suppressed in LUAD. **(A–F)** GSEA analysis in TCGA and GEO datasets based on Hallmark gene set.

GSEA analysis was carried out based on the Hallmark gene set to investigate the critical regulatory signaling pathways during tumorigenesis. We revealed that the inflammatory response pathway is suppressed in LUAD from TCGA (Figure 1A) which was verified in five GEO datasets (Figures 1B–F).

### Establishment of the IRRG Prognostic Signature

**FIGURE 2:**
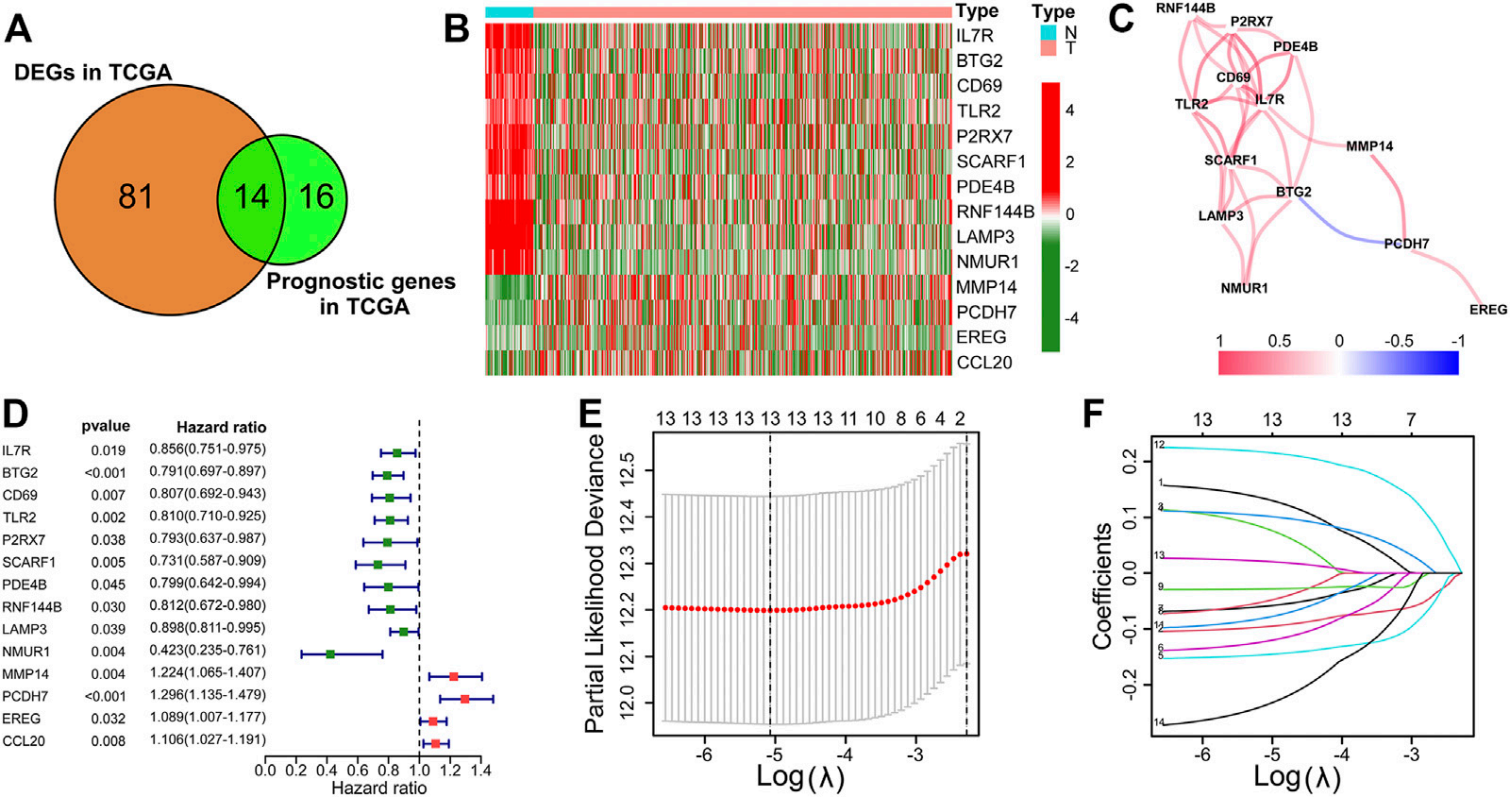
Establishment of the IRRG prognostic signature. **(A)** The Venn diagram shows 14 candidate intersection genes with differential expression and OS. **(B)** The 14 candidate genes’ expression heatmap in LUAD and normal samples. **(C)** The correlation analysis of 14 genes. **(D)** The forest plot of univariate Cox regression analysis of 14 genes. **(E)** LASSO regression with ten-fold cross-validation obtained 13 prognostic genes using the minimum λ. **(F)** LASSO coefficient profiles of 13 prognostic genes of LUAD.

We next investigated the differential expression analysis and survival analysis of 200 IRRG in TCGA. 14 genes presented differential expression and overall survival (Figure 2A). The heatmap of expression levels and forest plot of univariate COX regression analysis of these 14 genes are separately presented in Figures 2B,D. The relevance of the above prognostic genes is shown in Figure 2C. Then, we performed the LASSO algorithm to minimize the risk of overfitting, and 13 genes were reserved (Figures 2E,F). Ultimately, seven genes (MMP14, BTG2, LAMP3, CCL20, TLR2, IL7R, and PCDH7) were identified to construct an IRRG prognostic model using multivariate Cox regression analysis.

### Assessment and Validation of the IRRG Model

**FIGURE 3:**
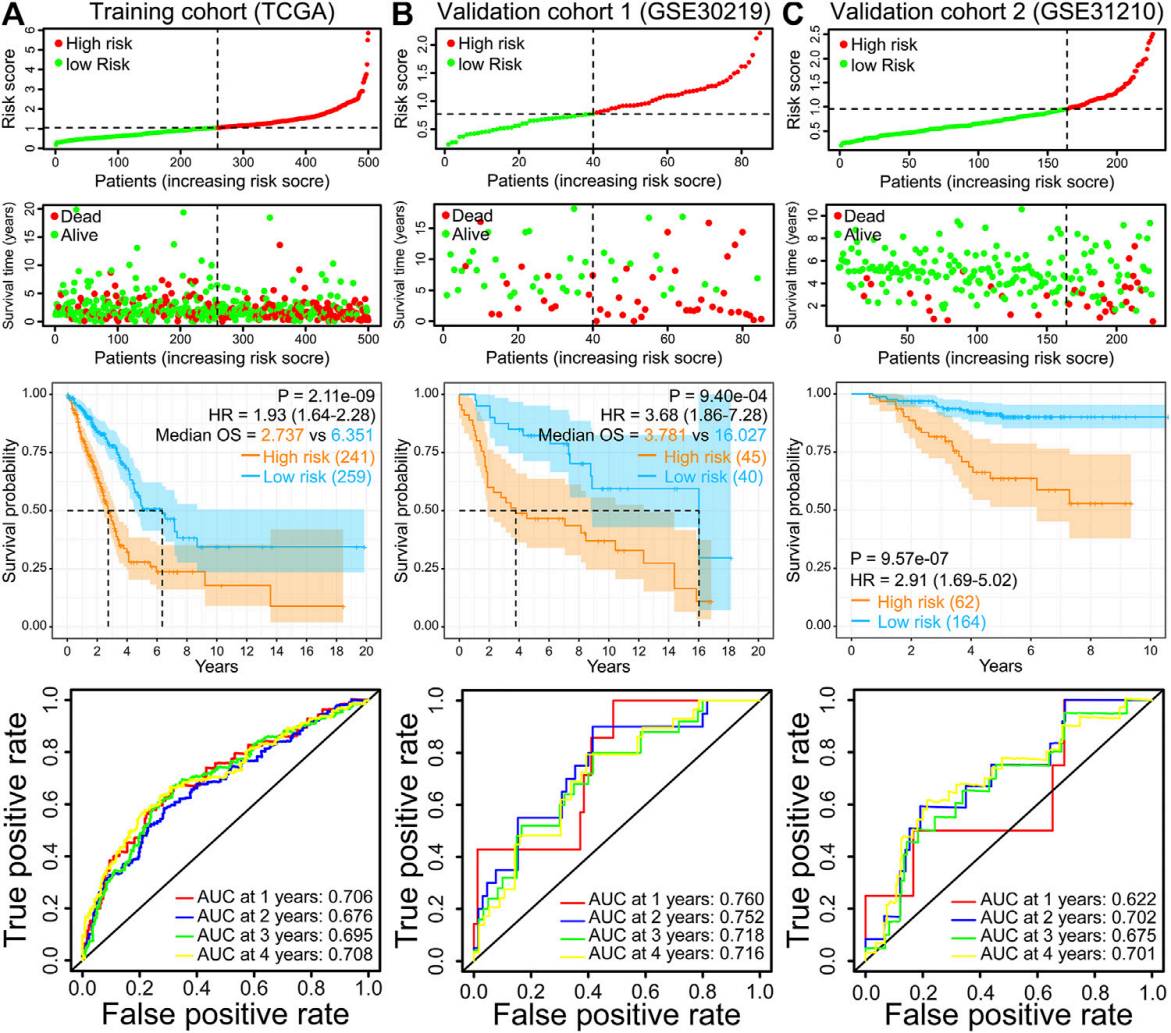
Risk score distribution, scatterplots, survival curve, and ROC curves of the IRRG prognostic model in the TCGA training cohort **(A)**, GSE30219 validation cohort **(B)**, and GSE31210 validation cohort **(C)**.

500 patients were split into two groups based on the best cut-off value in the training cohort (TCGA-LUAD). The risk score distribution, scatterplots, survival curves, and ROC curves of the training cohort are shown in Figure 3A. We demonstrated that the number of deaths in the high-risk group of the training cohort was markedly increased compared with that in the low-risk group (Figure 3A). The survival curve suggested that the overall survival time of high-risk groups was dramatically shorter than that of the low-risk group (Figure 3A). These results were also verified in GSE30219 and GSE31210 validation cohorts (Figures 3B,C). ROC curves in the training cohort demonstrated that the AUC values for 1-, 2-, 3-, and 4-year OS were 0.706, 0.676, 0.695, and 0.708, respectively (Figure 3A). The AUC for 4-year OS was as high as 0.716 and 0.701 in the GSE30219 and GSE31210 validation cohorts, respectively (Figures 3B,C).

**FIGURE 4:**
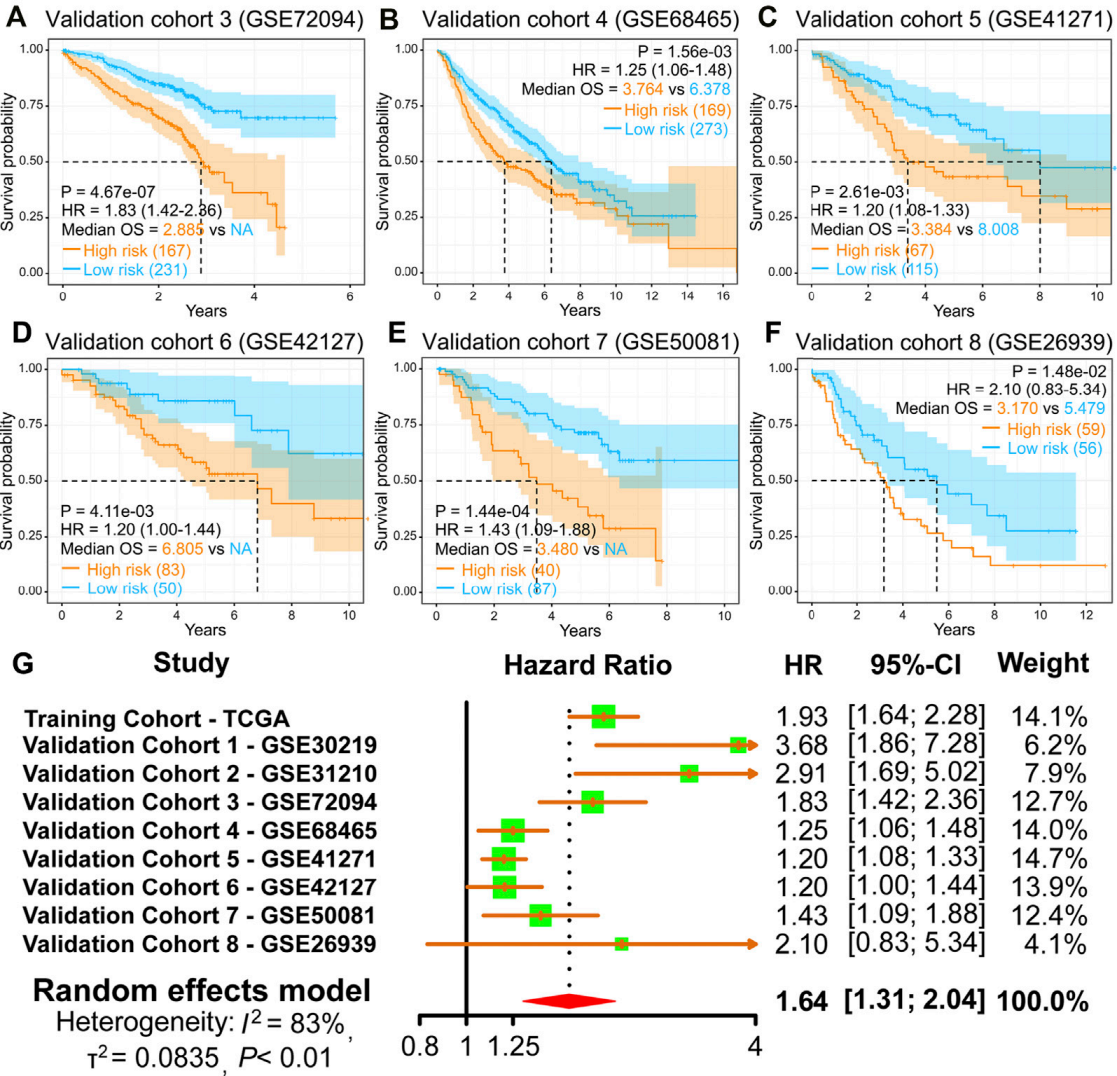
Validation of the IRRG prognostic model in LUAD from diverse GEO cohorts. **(A–F)** Survival curves of the IRRG prognostic model in six independent GEO cohorts. **(G)** Meta-analysis was executed to integrate HR values acquired from TCGA and eight GEO cohorts.

Moreover, the finding of the high-risk group predicting a worse OS was also verified in the other six GEO validation cohorts (GSE72094, GSE68465, GSE41271, GSE42127, GSE50081, and GSE26939) (Figures 4A–F). Moreover, we performed a meta-analysis to appraise the prognostic value by integrating TCGA and eight GEO cohorts. The meta-analysis results demonstrated that the pooled hazard ratio for the relevance between the high-risk score and OS was 1.64 (1.31–2.04) (Figure 4G). The above results manifest that the IRRG model performed well for OS prediction.

### Independent Prognostic Analysis

**FIGURE 5:**
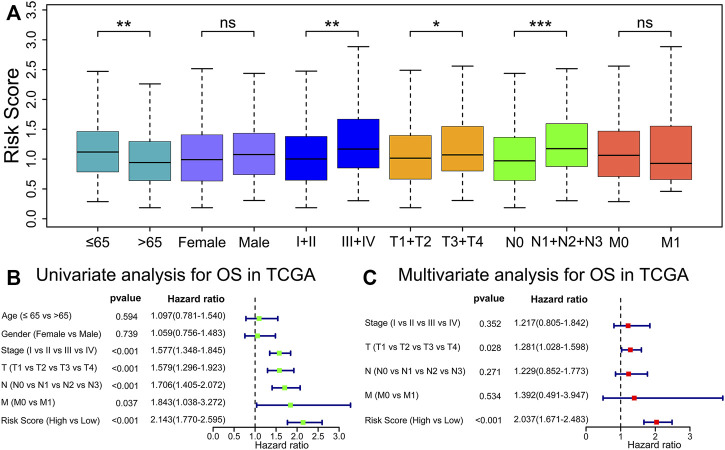
Risk score is an independent prognostic indicator for OS. **(A)** Comparisons of risk score among diverse clinicopathologic subgroups in the TCGA cohorts. ^*^ *p* < 0.05; ^**^ *p* < 0.01; ^***^ *p* < 0.001; ns, *p* ≥ 0.05. **(B,C)** Univariate and multivariable Cox regression analyses of risk score and different clinicopathological features.

We discovered that the risk score presented an increased value in tumor stages III–IV (*p* < 0.01), T stages 3–4 (*p* < 0.05), or N stages 1–3 (*p* < 0.001) than tumor stages I–II, T stages 1–2, or N stage 0 in TCGA cohort (Figure 5A). Next, we carried out univariate and multivariate Cox analyses in the TCGA cohort to evaluate whether the prognostic model risk score was an independent prognostic factor for overall survival. We found that the risk score presented a significant statistical difference in both univariate and multivariate Cox regression (Figures 5B,C), which indicates that the risk score is an independent prognostic indicator for overall survival.

### Nomogram Prediction Model Construction

**FIGURE 6:**
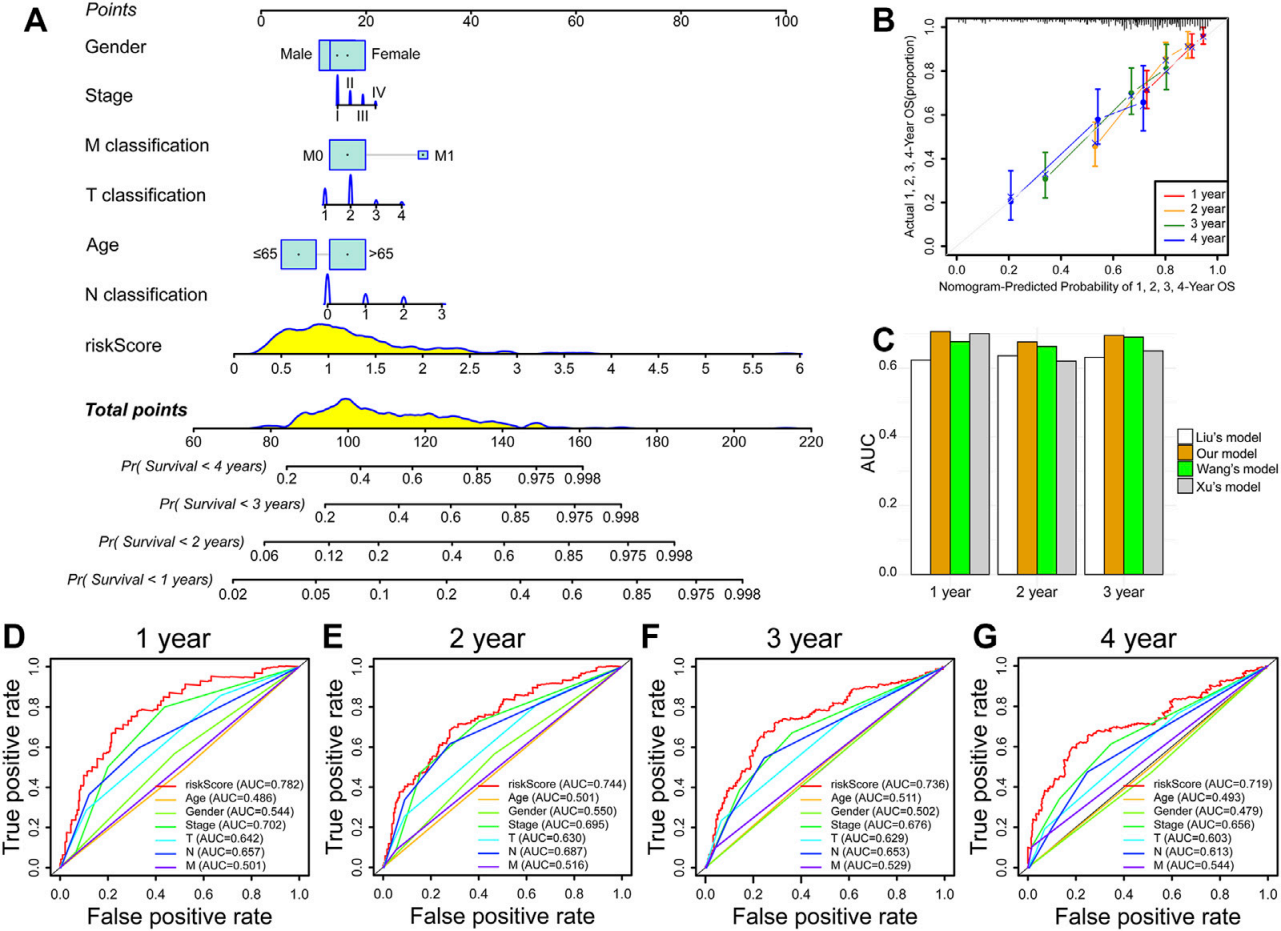
Nomogram prediction model construction. **(A)** The nomogram consists of risk score, age, gender, stage, T classification, N classification, and M classification. **(B)** Calibration curves of 1-, 2-, 3-, and 4-year OS prediction. **(C)** Comparison of AUC values in forecasting 1-, 2-, and 3-year survival between our and others’ models. **(D–G)** ROC curves of risk score, age, gender, stage, T classification, N classification, and M classification.

Moreover, we constructed a prognostic nomogram integrated with a risk score and multiple clinicopathologic characteristics to forecast patient prognosis accurately (Figure 6A). Interestingly, we found that risk score presented the greatest impact on prognosis forecast (Figure 6A). Calibration curves revealed satisfactory agreements between the nomogram forecast and the actual observations in the survival probability of 1, 2, 3, and 4 years (Figure 6B). Compared with other prognostic models (Liu T et al., 2021; Wang et al., 2021; Xu and Chen, 2021), the AUC value of our model is higher than others in forecasting 1-, 2-, and 3-year OS (Figure 6C). Meanwhile, the AUC of the prognostic model risk score was markedly higher than the AUC of the other clinicopathologic characteristics (Figures 6D–G). These results suggest that the IRRG model exhibits satisfactory accuracy and reliability.

### Identification of the Relationship Between Risk Score and Immune Infiltration Patterns

**FIGURE 7:**
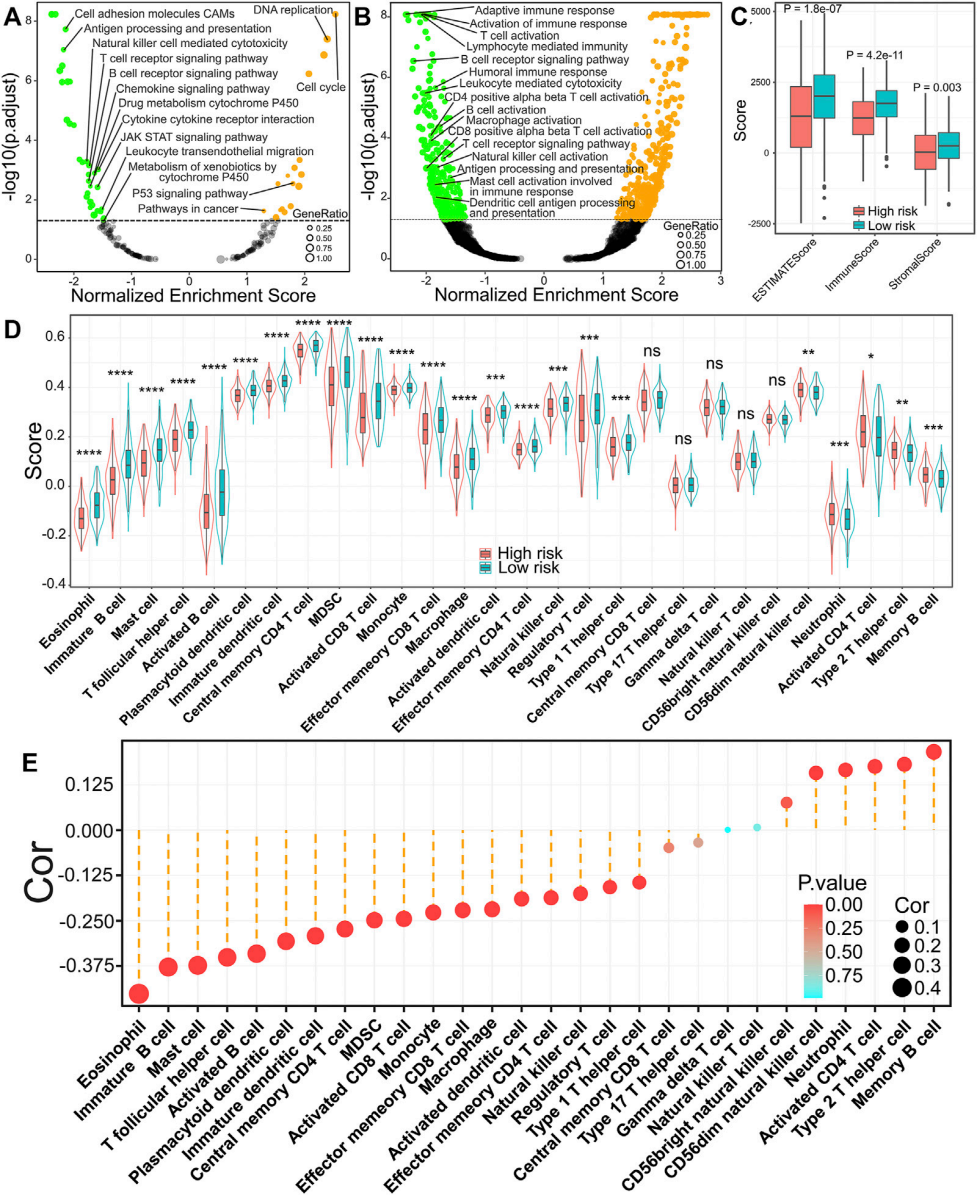
Relationship of the risk score with immune infiltration patterns in LUAD. **(A)** GSEA analysis for pathways in the KEGG database. **(B)** GSEA analysis for biological process in GO database. **(C)** Comparisons of ESTIMATE score, immune score, and stromal score between high- and low-risk groups. **(D)** The difference of 28 kinds of immune cells in high- or low-risk groups. ^*^ *p* < 0.05; ^**^ *p* < 0.01; ^***^ *p* < 0.001; ^****^ *p* < 0.0001; ns, *p* ≥ 0.05. **(E)** The relevance between risk score and 28 kinds of immune cells.

To further confirm the underlying biological mechanisms resulting in differential prognosis between high- and low-risk groups of the IRRG model, we carried out GSEA analysis according to KEGG and GO: BP gene sets in the TCGA cohort. These gene sets, such as cell cycle, DNA replication, the P53 signaling pathway, and so on, were activated in high-risk groups (Figure 7A). Interestingly, multiple gene sets were found to be associated with immune response, including antigen processing and presentation, natural killer cell-mediated cytotoxicity, the T cell receptor signaling pathway, the B cell receptor signaling pathway, and so on, were suppressed in high-risk groups (Figure 7A), which was verified in GSEA analysis for biological process in the GO database (Figure 7B). We further compared the immune, stromal, and ESTIMATE scores to explore the immune status between high- and low-risk groups. The results demonstrated that immune, stromal, and ESTIMATE scores were markedly lower in high-risk groups (Figure 7C). The above results indicate that tumor immune infiltration may be implicated in the dismal prognosis of high-risk groups.

**FIGURE 8:**
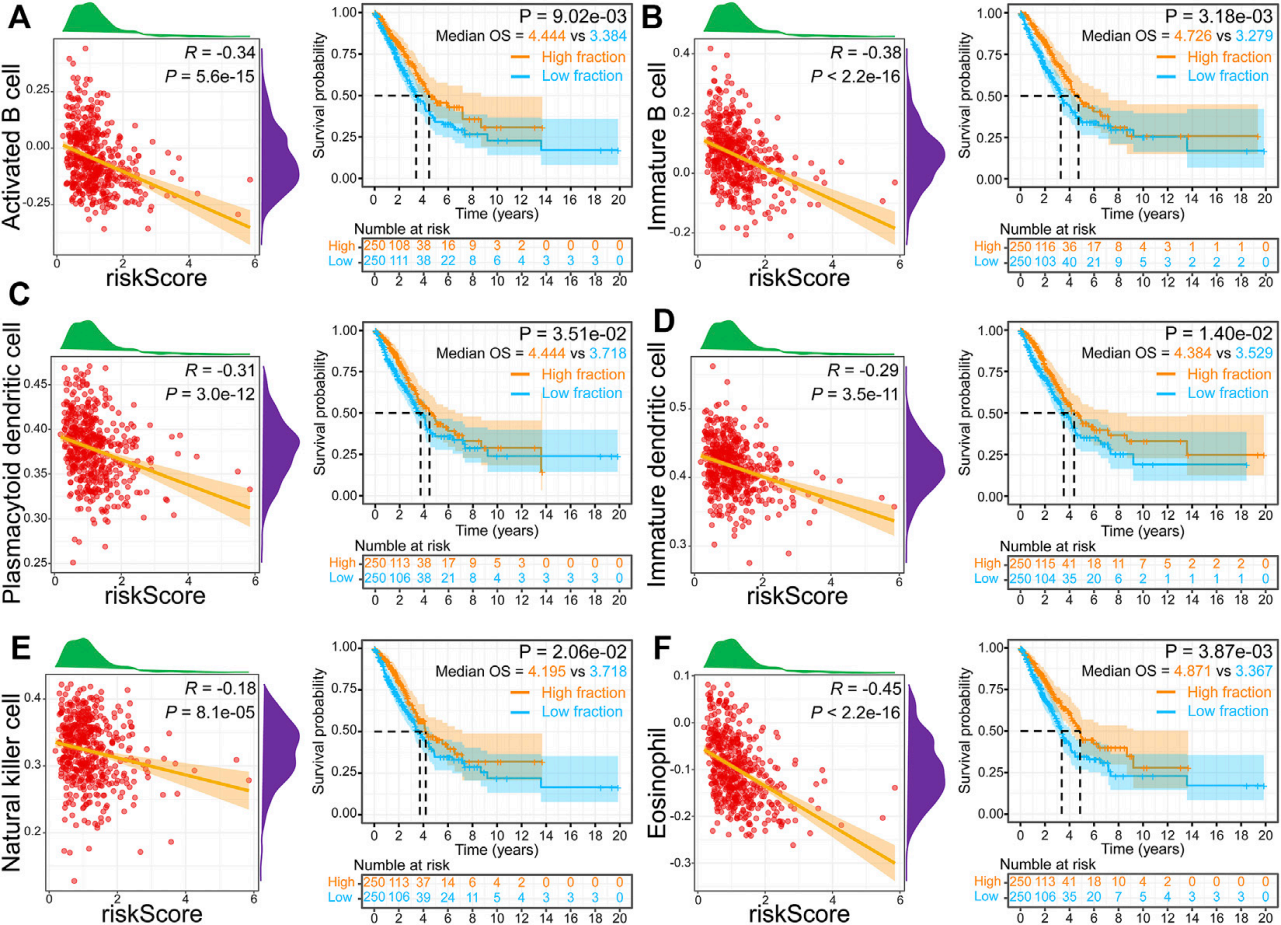
Relationship and survival curves between risk score and activated B cell **(A)**, immature B cell **(B)**, plasmacytoid dendritic cell **(C)**, immature dendritic cell **(D)**, natural killer cell **(E)**, and eosinophil **(F)**.

To better elucidate the relevance between risk score and tumor-infiltrating immune cells, we performed ssGSEA analysis. The infiltration levels of 18 immune cells were markedly reduced in high-risk groups. In comparison, five immune cells (CD56 bright natural killer cell, neutrophil, activated cd4 T cell, type 2 T helper cell, and memory B cell) were markedly increased in high-risk groups (Figure 7D). The correlation analysis results illuminated that the risk score was inversely correlated with 18 of 28 immune cells, while it was positively related to six immune cells (CD56 dim natural killer cell, CD56 bright natural killer cell, neutrophil, activated cd4 T cell, type 2 T helper cell, and memory B cell) (Figure 7E). Furthermore, we found that activated B cells (Figure 8A), immature B cells (Figure 8B), plasmacytoid dendritic cells (Figure 8C), immature dendritic cells (Figure 8D), natural killer cells (Figure 8E), and eosinophils (Figure 8F) were inversely associated with the risk score, while the low infiltration level of them was implicated in poor overall survival in LUAD. These results suggest that the above six immune cells can significantly affect the unsatisfactory prognostic of the high-risk group in LUAD.

### The Relationship Between Risk Score, Model Genes, and Chemosensitivity

**FIGURE 9:**
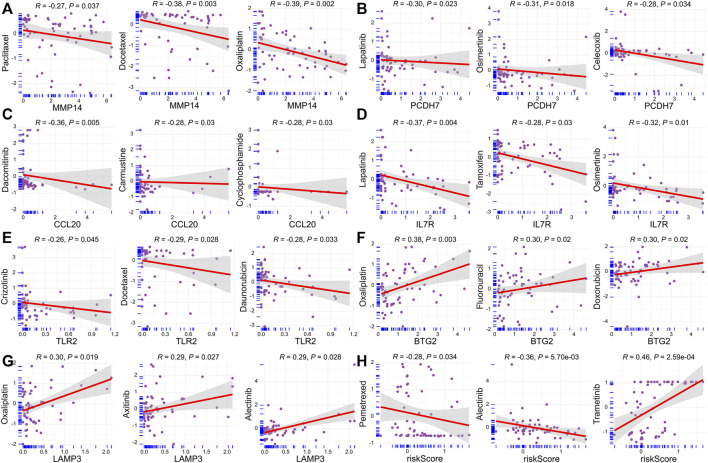
Relationship between risk score, model genes, and chemosensitivity. **(A)** MMP14. **(B)** PCDH7. **(C)** CCL20. **(D)** IL7R. **(E)** TLR2. **(F)** BTG2. **(G)** LAMP3. **(H)** Risk score.

We next performed the relationship analysis between risk score, model genes, and chemosensitivity using the Spearman method. We demonstrated that the high expression of MMP14, PCDH7, CCL20, IL7R, and TLR2 was inversely associated with the sensitivity of some commonly prescribed chemotherapeutic and targeted agents, including docetaxel, paclitaxel, crizotinib, osimertinib, etc (Figures 9A–E). In contrast, increased BTG2 and LAMP3 expression are accompanied by increased sensitivity of tumor cells to chemotherapeutic agents, such as axitinib, oxaliplatin, and fluorouracil (Figures 9F,G). Interestingly, we found that the risk score was inversely related to the drug sensitivity of pemetrexed and alectinib and positively associated with trametinib (Figure 9H).

### The Expression Validation of Model Genes

**FIGURE 10:**
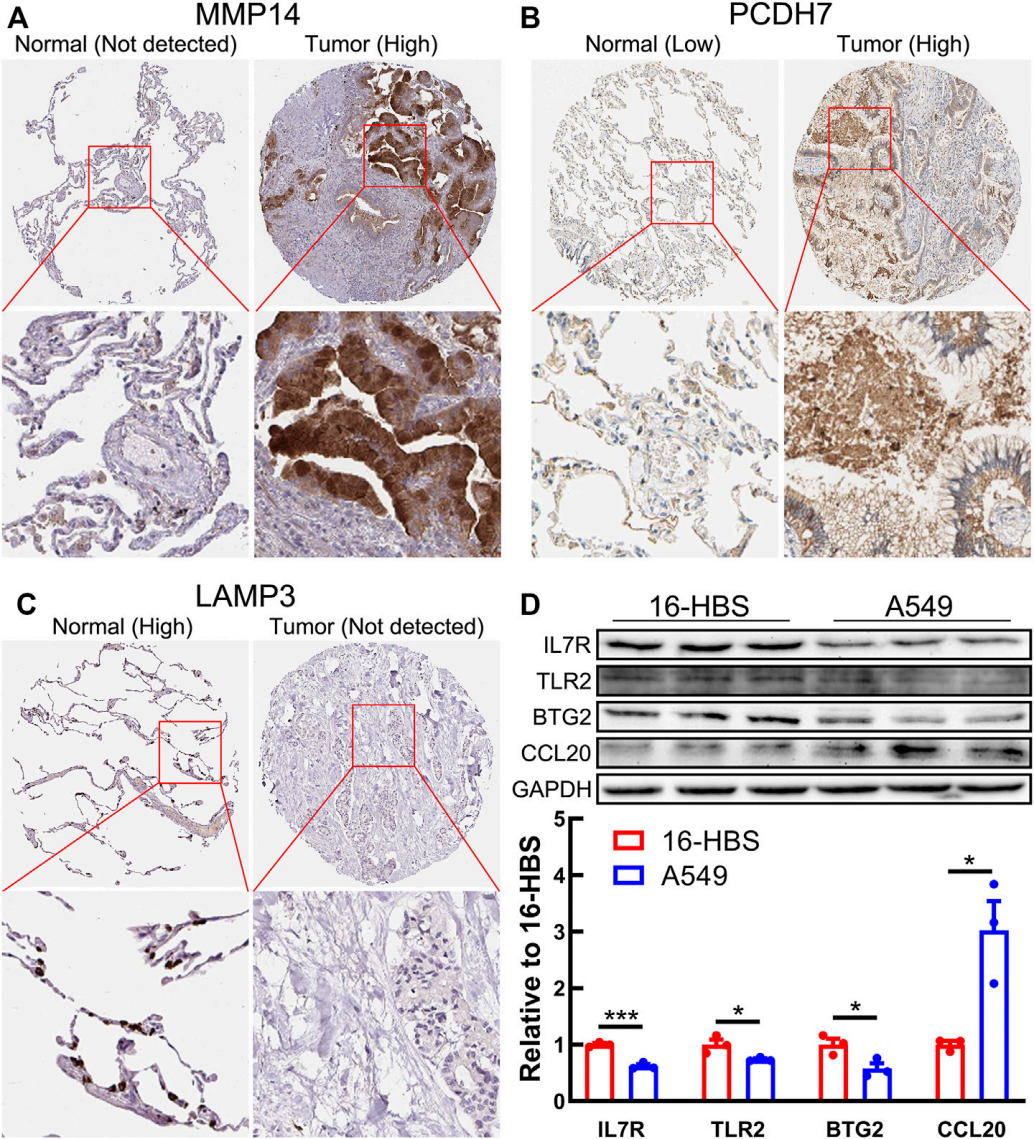
Protein expression of seven model genes. **(A–C)** Immunohistochemical staining images of MMP14 **(A)**, PCDH7 **(B)**, and LAMP3 **(C)** from the HPA database. **(D)** Western blotting results shows the protein expression of BTG2, TLR2, CCL20, and IL7R in a normal bronchial epithelial cell line (16-HBS) and lung adenocarcinoma cell line (A549).

Next, we performed the protein expression verification of the seven model genes above. Immunohistochemical staining results from the HPA database indicated that the protein expression of MMP14 and PCDH7 was significantly upregulated in LUAD tissues, while LAMP3 was significantly downregulated (Figures 10A–C). The results of Western blotting showed that BTG2, TLR2, and IL7R proteins were markedly downregulated in lung adenocarcinoma cell line A549, while CCL20 protein was markedly upregulated (Figure 10D).

## Discussion

Lung cancer morbidity has dropped to third, but mortality still ranks first (Siegel et al., 2021). With the advancement of sequencing and microarray technologies, increased attention has been focused on the impact of differentially expressed genes on the prognosis of lung cancer patients (Xu et al., 2021). The development of an accurate and reliable prognosis prediction tool based on differentially expressed genes is vital for therapeutic decision-making and prognostic assessment of LUAD patients (Xu and Chen, 2021). We demonstrated that the inflammatory response pathway is suppressed in LUAD tissues from TCGA and five GEO datasets compared with normal tissues. However, the relevance between IRRG signature and LUAD prognosis remains largely unknown.

This research established a robust 7-gene prognostic signature (MMP14, BTG2, LAMP3, CCL20, TLR2, IL7R, and PCDH7). Our prognostic model presented excellent and accurate forecasting ability in TCGA training cohort and eight GEO validation cohorts. The meta-analysis result also suggests that the risk score is an adverse factor for LUAD prognosis. Furthermore, the prognostic model risk score can act as a prognostic biomarker independent of diverse clinicopathological characteristics. The ROC curves demonstrated the superiority of risk score for prognostic assessment of LUAD patients than other clinical characteristics.

In order to increase our understanding of underlying biological mechanisms resulting in differential prognosis between high- and low-risk groups of the IRRG model, we carried out a GSEA analysis. Cell cycle and DNA replication that are thought to facilitate cancer progression (Matthews et al., 2021; Liu J et al., 2021) were activated in high-risk groups, whereas multiple gene sets related to immune response were suppressed in high-risk groups. Further analysis based on the ESTIMATE algorithm showed that immune scores were markedly lower in high-risk groups. These findings indicate that the survival difference among the two groups may be tightly associated with the tumor immune infiltration.

Based on the ssGSEA algorithm, lower B cell, dendritic cell, natural killer cell, and eosinophil infiltration level was inversely associated with the high-risk score and more prolonged overall survival in LUAD, which may likely explain the poor survival outcome in the high-risk group. Research has demonstrated that activated B cells directly *in vivo* present effective tumor inhibition (Wang S. S et al., 2019). Increased B cell infiltration was positively related to satisfactory prognosis in non–small-cell lung cancer (Federico et al., 2021). Meanwhile, B cell was an independent prognostic indicator for LUAD patients (Liu X et al., 2021). Dendritic cell is a professional antigen-presenting immune cell and contributes to a powerful anticancer immune response (van der Hoorn et al., 2021). However, the dendritic cell function is often suppressed in patients with lung cancer (Ma et al., 2021). Hence, some clinical trials are investigating neoantigen-targeted dendritic cell vaccines to promote antitumor immunity (Stevens et al., 2020). Natural killer cell can significantly affect tumor immunosurveillance by directly eliminating tumor cells and influencing metastasis by killing circulating cancer cells (Russick et al., 2020). Moreover, natural killer cell serves as a critical regulator for the recruitment and retention of dendritic cell (Ahluwalia et al., 2021). The role of eosinophils in tumors is controversial (Guerra et al., 2020). Interestingly, recent studies have found that eosinophils have antitumor activity in lung metastases and have anti-metastatic functions (Grisaru-Tal et al., 2021; Schuijs et al., 2020). In a word, the immune cells above are potentially involved in the regulation of immune response in LUAD development, leading to a poor survival outcome in the high-risk group.

Furthermore, we demonstrated that the tumor cells’ sensitivity to anticancer drugs was markedly related to risk scores and model genes. It is well known that pemetrexed is the first-line chemotherapy drug for lung adenocarcinoma. Interestingly, we found that the risk score was inversely related to the drug sensitivity of pemetrexed and alectinib and positively related to trametinib. In other words, these high-risk LUAD patients may not be candidates for the pemetrexed doublet chemotherapy, and trametinib may be an appropriate treatment for them.

Finally, we verified the protein expression of seven model genes using immunohistochemical staining images from the HPA database or Western blotting. We found that the protein expression of seven model genes was consistent with the mRNA expression. Interestingly, previous studies have demonstrated that these model genes are tightly implicated in the initiation and progression of lung carcinoma. For instance, the high expression of MMP14 (Stawowczyk et al., 2017) and CCL20 (Wang et al., 2016) facilitates the cancer cell proliferation and metastasis of lung carcinoma. BTG2 (Shen et al., 2018), IL7R (Fan et al., 2021), and LAMP3 (Lindskog et al., 2014) are significantly downregulated in carcinoma samples, and their high expressions are markedly implicated with more prolonged overall survival, which is consistent with our study. PCDH7 can act as an independent lung cancer prognosis marker and a potential therapeutic target (Zhou et al., 2019; Chen et al., 2021). The activation of TLR2 contributes to lung tumor progression by promoting the secretion of immune-suppressive cytokines (Pinto et al., 2011). These findings indicate the great potential of IRRG in the prognosis of lung cancer.

This study also has several limitations. First, the GSEA analysis result discovered that multiple pathways, such as glycolysis, TNFA signaling *via* NFKB, MYC targets V1, E2F targets, G2M checkpoint, and myogenesis, were activated or suppressed in six databases in LUAD tissues. The prognostic significance of these pathways in LUAD warrants further investigation. Second, studies have shown that TLR2 is highly expressed in LUAD (Gergen et al., 2020; Pu et al., 2021). However, our study found it to be downregulated in the A549 cell line. This is inconsistent. Further validation with more cell lines and more samples may clarify this question.

## Conclusion

The present study has demonstrated that the risk score of the IRRG model serves as an effective prognostic biomarker in LUAD. The model had good accuracy and reliability to discriminate high-risk patients in eight validation cohorts. Additionally, we found that low B cell, dendritic cell, natural killer cell, and eosinophil infiltration levels are responsible for poor prognosis in the high-risk group. However, the concrete molecular mechanism of survival discrepancy between high- and low-risk groups requires further biomedical experiments.

## Data Availability

The original contributions presented in the study are included in the article/Supplementary Material, further inquiries can be directed to the corresponding authors.

## Supplementary Material

Supplementary Table S1 200 inflammatory response–related genes.

Supplementary Table S2 792 chemotherapeutic or targeted drugs have received FDA approval or are currently in clinical trials.

Supplementary Table S3 All R codes.

## Abbreviations

GEO, Gene Expression Omnibus; ssGSEA, single sample gene set enrichment analysis; KM, Kaplan–Meier; LUAD, lung adenocarcinoma; OS, overall survival; IRRG, inflammatory response–related genes; TCGA, The Cancer Genome Atlas.
