# Supplementary material for: Development and Validation of a 7-Gene Inflammatory Signature Forecasts Prognosis and Diverse Immune Landscape in Lung Adenocarcinoma
Source: Front Mol Biosci. 2022 Mar 15;9:822739. doi: 10.3389/fmolb.2022.822739 (PMC8964604; doi:10.3389/fmolb.2022.822739)
Supplement: Supplementary file 1 [file DataSheet3.DOCX]

####

#### Results 1A

####

rm(list = ls())

exprSet <- data.table::fread("TCGA-LUAD.htseq_fpkm.txt", data.table = F)

test <- exprSet[1:50,1:20]

gtf1 <- rtracklayer::import('gencode.v22.annotation.gtf')

gtf_df <- as.data.frame(gtf1)

test <- gtf_df[1:10,1:27]

gtf_df1 <- gtf_df[,c(10,13,11,7)]

colnames(exprSet)[1] <- "gene_id"

library(dplyr)

exprSet1 <- gtf_df1 %>%

dplyr::filter(type=="gene",gene_type=="protein_coding") %>%

dplyr::select(c(gene_name,gene_id)) %>%

dplyr::inner_join(exprSet,by ="gene_id") %>%

dplyr::select(-gene_id) %>%

mutate(rowMean = rowMeans(.[,-1])) %>%

arrange(desc(rowMean)) %>%

distinct(gene_name,.keep_all = T) %>%

dplyr::select(-rowMean)

test1 <- exprSet1[1:10,1:10]

rownames(exprSet1) <- exprSet1$gene_name

exprSet1 <-exprSet1[,-1]

####

#### Delete duplicate samples (13 duplicate samples)

####

exprSet2 <- exprSet1 %>%

dplyr::select(-c("TCGA-44-2662-01B","TCGA-44-2666-01B","TCGA-44-2668-01B","TCGA-44-3917-01B","TCGA-44-3918-01B",

"TCGA-44-4112-01B","TCGA-44-5645-01B","TCGA-44-6146-01B","TCGA-44-6147-01B","TCGA-44-6775-01C",

"TCGA-50-5066-02A","TCGA-50-5946-02A","TCGA-44-2656-01B"))

test2 <- exprSet2[1:50,1:50]

####

exprSet3 <- as.data.frame(t(exprSet2))

metadata <- data.frame(rownames(exprSet3))

for (i in 1:nrow(metadata)) {

num <- as.numeric(substring(metadata[i,1],14,15))

if (num %in% seq(1,9)) {

metadata[i,2] <- "Tumor"

}

if (num %in% seq(10,29)) {

metadata[i,2] <- "Normal"

}

}

names(metadata) <- c("probe_id","sample")

table(metadata$sample)

exprSet4 <- exprSet3 %>%

rownames_to_column("probe_id") %>%

inner_join(metadata,by="probe_id") %>%

select(probe_id,sample,everything())

rownames(exprSet4) <- exprSet4$probe_id

exprSet4 <-exprSet4[,-1]

test4 <- exprSet4[,1:5]

table(exprSet4$sample)

Normal <- exprSet4[exprSet4$sample %in% c("Normal"),]

Tumor <- exprSet4[exprSet4$sample %in% c("Tumor"),]

exprSet1 <- rbind(Normal, Tumor)

save(exprSet1, file = "TCGA_LUAD_59Normal_513Tumor.Rdata")

####

#### Bayes test

####

rm(list = ls())

load("TCGA_LUAD_8_59Normal_513Tumor.Rdata")

test <- exprSet1[1:50,1:20]

library(limma)

exprSet <- as.data.frame(t(exprSet1[,-1]))

test <- exprSet[1:50,1:50]

group <- c(rep("Normal",59),rep("Tumor",513))

group <- factor(group,levels = c("Normal","Tumor"),ordered = F)

design <- model.matrix(~group)

colnames(design) <- levels(group)

fit <- lmFit(exprSet,design)

fit2 <- eBayes(fit)

all_Diff_gene = topTable(fit2,adjust='fdr',coef=2,number=Inf)

save(all_Diff_gene, file = "TCGA_all_Diff_gene.Rdata")

rt <- all_Diff_gene

rt$logFC <- as.numeric(rt$logFC)

rt$adj.P.Val <- as.numeric(rt$adj.P.Val)

library(tibble)

library(dplyr)

rt <- rt %>%

rownames_to_column("gene")

colnames(rt)[2] <- c("logFC")

gene <- rt$gene

library(clusterProfiler)

gene = bitr(gene, fromType="SYMBOL", toType="ENTREZID", OrgDb="org.Hs.eg.db")

gene <- dplyr::distinct(gene,SYMBOL,.keep_all=TRUE)

gene_df <- data.frame(logFC=rt$logFC,SYMBOL = rt$gene)

gene_df <- merge(gene_df,gene,by="SYMBOL")

colnames(gene_df) <- c("gene","logFC","entrez")

geneList <- gene_df$logFC

names(geneList) = gene_df$gene

geneList = sort(geneList, decreasing = TRUE)

head(geneList)

####

#### Hallmarks GSEA

####

library(clusterProfiler)

hallmarks <- read.gmt("h.all.v7.4.symbols.gmt")

gseahallmarks <- GSEA(geneList,TERM2GENE =hallmarks)

library(ggplot2)

dotplot(gseahallmarks,showCategory=5,split=".sign")+facet_grid(~.sign)

library(export)

graph2ppt(file= "Results_1A.ppt", width=7, height=5)

####

#### Results 1B

####

rm(list = ls())

dd1 <- data.table::fread("GSE75037_series_matrix.txt",skip= 81,data.table = F)

test <- dd1[1:10,1:10]

rownames(dd1) <- dd1[,1]

exprSet <- dd1[,-1]

boxplot(exprSet,outline=FALSE, notch=T, las=2)

ex <- exprSet

qx <- as.numeric(quantile(ex, c(0., 0.25, 0.5, 0.75, 0.99, 1.0), na.rm=T))

LogC <- (qx[5] > 100) ||

(qx[6]-qx[1] > 50 && qx[2] > 0) ||

(qx[2] > 0 && qx[2] < 1 && qx[4] > 1 && qx[4] < 2)

if (LogC) {

ex[which(ex <= 0)] <- NaN

exprSet <- log2(ex)

print("log2 transform finished")

}else{

print("log2 transform not needed")

}

library(limma)

exprSet=normalizeBetweenArrays(exprSet)

boxplot(exprSet,outline=FALSE, notch=T, las=2)

exprSet <- as.data.frame(exprSet)

####

GEO_soft <-data.table::fread("GSE75037_family.soft",skip = 266,data.table=F)

GEO_soft1 <-GEO_soft[,-c(2:12,14:28)]

colnames(GEO_soft1)[1:2] <- c("probe_id","GENE_SYMBOL")

length(unique(GEO_soft1$GENE_SYMBOL))

library(dplyr)

library(tibble)

exprSet1 <- exprSet %>%

rownames_to_column("probe_id") %>%

inner_join(GEO_soft1,by="probe_id") %>%

dplyr::select(-probe_id) %>%

dplyr::select(GENE_SYMBOL,everything()) %>%

mutate(rowMean =rowMeans(.[,-1])) %>%

arrange(desc(rowMean)) %>%

distinct(GENE_SYMBOL,.keep_all = T) %>%

dplyr::select(-rowMean) %>%

column_to_rownames("GENE_SYMBOL")

test1 <- exprSet1[1:10,1:10]

exprSet2 <- exprSet1[,c(seq(1,166,by=2),seq(2,166,by=2))]

test2 <- exprSet2[1:10,1:10]

####

#### Bayes test

####

library(limma)

exprSet <- as.data.frame(exprSet2)

group <- c(rep("Control",83),rep("Tumor",83))

group <- factor(group,levels = c("Control","Tumor"),ordered = F)

design <- model.matrix(~group)

colnames(design) <- levels(group)

fit <- lmFit(exprSet,design)

fit2 <- eBayes(fit)

all_Diff_gene = topTable(fit2,adjust='fdr',coef=2,number=Inf)

rt <- all_Diff_gene

rt$logFC <- as.numeric(rt$logFC)

rt$adj.P.Val <- as.numeric(rt$adj.P.Val)

library(tibble)

library(dplyr)

rt <- rt %>%

rownames_to_column("gene")

colnames(rt)[2] <- c("logFC")

gene <- rt$gene

library(clusterProfiler)

gene = bitr(gene, fromType="SYMBOL", toType="ENTREZID", OrgDb="org.Hs.eg.db")

gene <- dplyr::distinct(gene,SYMBOL,.keep_all=TRUE)

gene_df <- data.frame(logFC=rt$logFC,SYMBOL = rt$gene)

gene_df <- merge(gene_df,gene,by="SYMBOL")

colnames(gene_df) <- c("gene","logFC","entrez")

geneList <- gene_df$logFC

names(geneList) = gene_df$gene

geneList = sort(geneList, decreasing = TRUE)

head(geneList)

####

#### Hallmarks GSEA

####

library(clusterProfiler)

hallmarks <- read.gmt("h.all.v7.4.symbols.gmt")

gseahallmarks <- GSEA(geneList,TERM2GENE =hallmarks)

library(ggplot2)

dotplot(gseahallmarks,showCategory=5,split=".sign")+facet_grid(~.sign)

library(export)

graph2ppt(file= "Results_1B.ppt", width=7.7, height=5)

####

#### Results 1C

####

rm(list = ls())

dd1 <- data.table::fread("GSE63459_series_matrix.txt",skip= 75,data.table = F)

test <- dd1[1:10,1:10]

rownames(dd1) <- dd1[,1]

exprSet <- dd1[,-1]

boxplot(exprSet,outline=FALSE, notch=T, las=2)

ex <- exprSet

qx <- as.numeric(quantile(ex, c(0., 0.25, 0.5, 0.75, 0.99, 1.0), na.rm=T))

LogC <- (qx[5] > 100) ||

(qx[6]-qx[1] > 50 && qx[2] > 0) ||

(qx[2] > 0 && qx[2] < 1 && qx[4] > 1 && qx[4] < 2)

if (LogC) {

ex[which(ex <= 0)] <- NaN

exprSet <- log2(ex)

print("log2 transform finished")

}else{

print("log2 transform not needed")

}

library(limma)

exprSet=normalizeBetweenArrays(exprSet)

boxplot(exprSet,outline=FALSE, notch=T, las=2)

exprSet <- as.data.frame(exprSet)

GEO_soft <-data.table::fread("GSE63459_family.soft",skip = 157,data.table=F)

GEO_soft1 <-GEO_soft[,-c(2:11,13:27)]

colnames(GEO_soft1)[1:2] <- c("probe_id","GENE_SYMBOL")

length(unique(GEO_soft1$GENE_SYMBOL))

library(dplyr)

library(tibble)

exprSet1 <- exprSet %>%

rownames_to_column("probe_id") %>%

inner_join(GEO_soft1,by="probe_id") %>%

dplyr::select(-probe_id) %>%

dplyr::select(GENE_SYMBOL,everything()) %>%

mutate(rowMean =rowMeans(.[,-1])) %>%

arrange(desc(rowMean)) %>%

distinct(GENE_SYMBOL,.keep_all = T) %>%

dplyr::select(-rowMean) %>%

column_to_rownames("GENE_SYMBOL")

test1 <- exprSet1[1:10,1:10]

exprSet2 <- exprSet1 %>%

dplyr::select(GSM1550170,GSM1550172,GSM1550174,GSM1550177,GSM1550179,GSM1550181,GSM1550183,GSM1550185,GSM1550187,GSM1550189,

GSM1550191,GSM1550194,GSM1550196,GSM1550198,GSM1550200,GSM1550202,GSM1550203,GSM1550205,GSM1550207,GSM1550209,

GSM1550211,GSM1550213,GSM1550215,GSM1550217,GSM1550219,GSM1550221,GSM1550223,GSM1550225,GSM1550227,GSM1550229,

GSM1550231,GSM1550233,everything())

test2 <- exprSet2[1:10,1:40]

####

#### Bayes test

####

library(limma)

exprSet <- as.data.frame(exprSet2)

group <- c(rep("Control",32),rep("Tumor",33))

group <- factor(group,levels = c("Control","Tumor"),ordered = F)

design <- model.matrix(~group)

colnames(design) <- levels(group)

fit <- lmFit(exprSet,design)

fit2 <- eBayes(fit)

all_Diff_gene = topTable(fit2,adjust='fdr',coef=2,number=Inf)

rt <- all_Diff_gene

rt$logFC <- as.numeric(rt$logFC)

rt$adj.P.Val <- as.numeric(rt$adj.P.Val)

library(tibble)

library(dplyr)

rt <- rt %>%

rownames_to_column("gene")

colnames(rt)[2] <- c("logFC")

gene <- rt$gene

library(clusterProfiler)

gene = bitr(gene, fromType="SYMBOL", toType="ENTREZID", OrgDb="org.Hs.eg.db")

gene <- dplyr::distinct(gene,SYMBOL,.keep_all=TRUE)

gene_df <- data.frame(logFC=rt$logFC,SYMBOL = rt$gene)

gene_df <- merge(gene_df,gene,by="SYMBOL")

colnames(gene_df) <- c("gene","logFC","entrez")

geneList <- gene_df$logFC

names(geneList) = gene_df$gene

geneList = sort(geneList, decreasing = TRUE)

head(geneList)

####

#### Hallmarks GSEA

####

library(clusterProfiler)

hallmarks <- read.gmt("h.all.v7.4.symbols.gmt")

gseahallmarks <- GSEA(geneList,TERM2GENE =hallmarks)

library(ggplot2)

dotplot(gseahallmarks,showCategory=5,split=".sign")+facet_grid(~.sign)

library(export)

graph2ppt(file= "Results_1C.ppt", width=7.2, height=5)

####

#### Results 1D

####

rm(list = ls())

dd1 <- data.table::fread("GSE43458_series_matrix.txt",skip= 58,data.table = F)

test <- dd1[1:10,1:10]

rownames(dd1) <- dd1[,1]

exprSet <- dd1[,-1]

boxplot(exprSet,outline=FALSE, notch=T, las=2)

ex <- exprSet

qx <- as.numeric(quantile(ex, c(0., 0.25, 0.5, 0.75, 0.99, 1.0), na.rm=T))

LogC <- (qx[5] > 100) ||

(qx[6]-qx[1] > 50 && qx[2] > 0) ||

(qx[2] > 0 && qx[2] < 1 && qx[4] > 1 && qx[4] < 2)

if (LogC) {

ex[which(ex <= 0)] <- NaN

exprSet <- log2(ex)

print("log2 transform finished")

}else{

print("log2 transform not needed")

}

library(limma)

exprSet=normalizeBetweenArrays(exprSet)

boxplot(exprSet,outline=FALSE, notch=T, las=2)

exprSet <- as.data.frame(exprSet)

####

options(BioC_mirror="https://mirrors.ustc.edu.cn/bioc/")

if(!require("hugene10sttranscriptcluster.db")) BiocManager::install("hugene10sttranscriptcluster.db",update = F,ask = F)

probe2symbol_df <- toTable(get("hugene10sttranscriptclusterSYMBOL"))

length(unique(probe2symbol_df$symbol))

nrow(probe2symbol_df)

library(dplyr)

library(tibble)

test <- exprSet[1:10,1:10]

exprSet1 <- exprSet %>%

rownames_to_column("probe_id") %>%

inner_join(probe2symbol_df,by="probe_id") %>%

dplyr::select(-probe_id) %>%

dplyr::select(symbol,everything()) %>%

mutate(rowMean =rowMeans(.[,-1])) %>%

arrange(desc(rowMean)) %>%

distinct(symbol,.keep_all = T) %>%

dplyr::select(-rowMean) %>%

column_to_rownames("symbol")

test1 <- exprSet1[1:10,1:10]

library(tibble)

exprSet2 <- exprSet1 %>%

select(GSM1062805,GSM1062806,GSM1062807,GSM1062808,GSM1062809,GSM1062810,GSM1062811,GSM1062812,

GSM1062813,GSM1062814,GSM1062815,GSM1062816,GSM1062817,GSM1062818,GSM1062819,GSM1062820,

GSM1062821,GSM1062822,GSM1062823,GSM1062824,GSM1062825,GSM1062826,GSM1062827,GSM1062828,

GSM1062829,GSM1062830,GSM1062831,GSM1062832,GSM1062833,GSM1062834,everything())

test2 <- exprSet2[1:10,1:50]

####

#### Bayes test

####

library(limma)

exprSet <- as.data.frame(exprSet2)

group <- c(rep("Control",30),rep("Tumor",80))

group <- factor(group,levels = c("Control","Tumor"),ordered = F)

design <- model.matrix(~group)

colnames(design) <- levels(group)

fit <- lmFit(exprSet,design)

fit2 <- eBayes(fit)

all_Diff_gene = topTable(fit2,adjust='fdr',coef=2,number=Inf)

rt <- all_Diff_gene

rt$logFC <- as.numeric(rt$logFC)

rt$adj.P.Val <- as.numeric(rt$adj.P.Val)

library(tibble)

library(dplyr)

rt <- rt %>%

rownames_to_column("gene")

colnames(rt)[2] <- c("logFC")

gene <- rt$gene

library(clusterProfiler)

gene = bitr(gene, fromType="SYMBOL", toType="ENTREZID", OrgDb="org.Hs.eg.db")

gene <- dplyr::distinct(gene,SYMBOL,.keep_all=TRUE)

gene_df <- data.frame(logFC=rt$logFC,SYMBOL = rt$gene)

gene_df <- merge(gene_df,gene,by="SYMBOL")

colnames(gene_df) <- c("gene","logFC","entrez")

geneList <- gene_df$logFC

names(geneList) = gene_df$gene

geneList = sort(geneList, decreasing = TRUE)

head(geneList)

####

#### Hallmarks GSEA

####

library(clusterProfiler)

hallmarks <- read.gmt("h.all.v7.4.symbols.gmt")

gseahallmarks <- GSEA(geneList,TERM2GENE =hallmarks)

library(ggplot2)

dotplot(gseahallmarks,showCategory=5,split=".sign")+facet_grid(~.sign)

library(export)

graph2ppt(file= "Results_1D.ppt", width=7.2, height=5)

####

#### Results 1E

####

rm(list = ls())

dd1 <- data.table::fread("GSE31210_series_matrix.txt",skip= 75,data.table = F)

test <- dd1[1:10,1:10]

rownames(dd1) <- dd1[,1]

exprSet <- dd1[,-1]

boxplot(exprSet,outline=FALSE, notch=T, las=2)

ex <- exprSet

qx <- as.numeric(quantile(ex, c(0., 0.25, 0.5, 0.75, 0.99, 1.0), na.rm=T))

LogC <- (qx[5] > 100) ||

(qx[6]-qx[1] > 50 && qx[2] > 0) ||

(qx[2] > 0 && qx[2] < 1 && qx[4] > 1 && qx[4] < 2)

if (LogC) {

ex[which(ex <= 0)] <- NaN

exprSet <- log2(ex)

print("log2 transform finished")

}else{

print("log2 transform not needed")

}

library(limma)

exprSet=normalizeBetweenArrays(exprSet)

boxplot(exprSet,outline=FALSE, notch=T, las=2)

exprSet <- as.data.frame(exprSet)

GEO_soft <-data.table::fread("GSE31210_family.soft",skip = 390,data.table=F)

GEO_soft1 <-GEO_soft[,-c(2:10,12:16)]

colnames(GEO_soft1)[1:2] <- c("probe_id","GENE_SYMBOL")

length(unique(GEO_soft1$GENE_SYMBOL))

library(dplyr)

library(tibble)

exprSet1 <- exprSet %>%

rownames_to_column("probe_id") %>%

inner_join(GEO_soft1,by="probe_id") %>%

dplyr::select(-probe_id) %>%

dplyr::select(GENE_SYMBOL,everything()) %>%

mutate(rowMean =rowMeans(.[,-1])) %>%

arrange(desc(rowMean)) %>%

distinct(GENE_SYMBOL,.keep_all = T) %>%

dplyr::select(-rowMean) %>%

column_to_rownames("GENE_SYMBOL")

test1 <- exprSet1[1:10,1:10]

library(tibble)

exprSet2 <- exprSet1 %>%

select(GSM773766,GSM773767,GSM773768,GSM773769,GSM773770,GSM773771,GSM773772,GSM773773,GSM773774,GSM773775,

GSM773776,GSM773777,GSM773778,GSM773779,GSM773780,GSM773781,GSM773782,GSM773783,GSM773784,GSM773785,everything())

test2 <- exprSet2[1:10,1:20]

####

#### Bayes test

####

library(limma)

exprSet <- as.data.frame(exprSet2)

group <- c(rep("Control",20),rep("Tumor",226))

group <- factor(group,levels = c("Control","Tumor"),ordered = F)

design <- model.matrix(~group)

colnames(design) <- levels(group)

fit <- lmFit(exprSet,design)

fit2 <- eBayes(fit)

all_Diff_gene = topTable(fit2,adjust='fdr',coef=2,number=Inf)

rt <- all_Diff_gene

rt$logFC <- as.numeric(rt$logFC)

rt$adj.P.Val <- as.numeric(rt$adj.P.Val)

library(tibble)

library(dplyr)

rt <- rt %>%

rownames_to_column("gene")

colnames(rt)[2] <- c("logFC")

gene <- rt$gene

library(clusterProfiler)

gene = bitr(gene, fromType="SYMBOL", toType="ENTREZID", OrgDb="org.Hs.eg.db")

gene <- dplyr::distinct(gene,SYMBOL,.keep_all=TRUE)

gene_df <- data.frame(logFC=rt$logFC,SYMBOL = rt$gene)

gene_df <- merge(gene_df,gene,by="SYMBOL")

colnames(gene_df) <- c("gene","logFC","entrez")

geneList <- gene_df$logFC

names(geneList) = gene_df$gene

geneList = sort(geneList, decreasing = TRUE)

head(geneList)

####

#### Hallmarks GSEA

####

library(clusterProfiler)

hallmarks <- read.gmt("h.all.v7.4.symbols.gmt")

gseahallmarks <- GSEA(geneList,TERM2GENE =hallmarks)

library(ggplot2)

dotplot(gseahallmarks,showCategory=5,split=".sign")+facet_grid(~.sign)

library(export)

graph2ppt(file= "Results_1F.pdf", width=7.2, height=5)

####

#### Results 1G

####

rm(list = ls())

dd1 <- data.table::fread("GSE30219_series_matrix.txt",skip= 84,data.table = F)

test <- dd1[1:10,1:10]

rownames(dd1) <- dd1[,1]

exprSet <- dd1[,-1]

boxplot(exprSet,outline=FALSE, notch=T, las=2)

ex <- exprSet

qx <- as.numeric(quantile(ex, c(0., 0.25, 0.5, 0.75, 0.99, 1.0), na.rm=T))

LogC <- (qx[5] > 100) ||

(qx[6]-qx[1] > 50 && qx[2] > 0) ||

(qx[2] > 0 && qx[2] < 1 && qx[4] > 1 && qx[4] < 2)

if (LogC) {

ex[which(ex <= 0)] <- NaN

exprSet <- log2(ex)

print("log2 transform finished")

}else{

print("log2 transform not needed")

}

library(limma)

exprSet=normalizeBetweenArrays(exprSet)

boxplot(exprSet,outline=FALSE, notch=T, las=2)

exprSet <- as.data.frame(exprSet)

GEO_soft <-data.table::fread("GPL570-55999.txt",skip = 16,data.table=F)

GEO_soft1 <-GEO_soft[,-c(2:10,12:16)]

colnames(GEO_soft1)[1:2] <- c("probe_id","GENE_SYMBOL")

length(unique(GEO_soft1$GENE_SYMBOL))

library(dplyr)

library(tibble)

exprSet1 <- exprSet %>%

rownames_to_column("probe_id") %>%

inner_join(GEO_soft1,by="probe_id") %>%

dplyr::select(-probe_id) %>%

dplyr::select(GENE_SYMBOL,everything()) %>%

mutate(rowMean =rowMeans(.[,-1])) %>%

arrange(desc(rowMean)) %>%

distinct(GENE_SYMBOL,.keep_all = T) %>%

dplyr::select(-rowMean) %>%

column_to_rownames("GENE_SYMBOL")

test1 <- exprSet1[1:10,1:10]

library(tibble)

exprSet2 <- exprSet1 %>%

select(GSM748060,GSM748070,GSM748107,GSM748118,GSM748129,GSM748153,GSM748158,GSM748168,GSM748176,GSM748184,

GSM748232,GSM748233,GSM748234,GSM748235,

GSM748053,GSM748054,GSM748055,GSM748057,GSM748058,GSM748064,GSM748068,GSM748071,GSM748075,

GSM748077,GSM748078,GSM748079,GSM748080,GSM748081,GSM748083,GSM748085,GSM748086,GSM748087,

GSM748089,GSM748090,GSM748091,GSM748092,GSM748094,GSM748100,GSM748101,GSM748102,GSM748103,

GSM748104,GSM748108,GSM748111,GSM748113,GSM748116,GSM748121,GSM748122,GSM748123,GSM748124,

GSM748125,GSM748126,GSM748127,GSM748128,GSM748130,GSM748131,GSM748134,GSM748135,GSM748137,

GSM748138,GSM748139,GSM748140,GSM748142,GSM748143,GSM748146,GSM748147,GSM748150,GSM748152,

GSM748157,GSM748161,GSM748162,GSM748164,GSM748166,GSM748167,GSM748169,GSM748170,GSM748171,

GSM748173,GSM748175,GSM748178,GSM748179,GSM748180,GSM748181,GSM748185,GSM748186,GSM748187,

GSM748188,GSM748189,GSM748239,GSM748240,GSM748242,GSM748243,GSM748264,GSM748271,GSM748276,

GSM748277,GSM748278,GSM748279,GSM1465989)

test2 <- exprSet2[1:10,1:50]

####

#### Bayes test

####

library(limma)

exprSet <- as.data.frame(exprSet2)

group <- c(rep("Control",14),rep("Tumor",85))

group <- factor(group,levels = c("Control","Tumor"),ordered = F)

design <- model.matrix(~group)

colnames(design) <- levels(group)

fit <- lmFit(exprSet,design)

fit2 <- eBayes(fit)

all_Diff_gene = topTable(fit2,adjust='fdr',coef=2,number=Inf)

rt <- all_Diff_gene

rt$logFC <- as.numeric(rt$logFC)

rt$adj.P.Val <- as.numeric(rt$adj.P.Val)

library(tibble)

library(dplyr)

rt <- rt %>%

rownames_to_column("gene")

colnames(rt)[2] <- c("logFC")

gene <- rt$gene

library(clusterProfiler)

gene = bitr(gene, fromType="SYMBOL", toType="ENTREZID", OrgDb="org.Hs.eg.db")

gene <- dplyr::distinct(gene,SYMBOL,.keep_all=TRUE)

gene_df <- data.frame(logFC=rt$logFC,SYMBOL = rt$gene)

gene_df <- merge(gene_df,gene,by="SYMBOL")

colnames(gene_df) <- c("gene","logFC","entrez")

geneList <- gene_df$logFC

names(geneList) = gene_df$gene

geneList = sort(geneList, decreasing = TRUE)

head(geneList)

####

#### Hallmarks GSEA

####

library(clusterProfiler)

hallmarks <- read.gmt("h.all.v7.4.symbols.gmt")

gseahallmarks <- GSEA(geneList,TERM2GENE =hallmarks)

library(ggplot2)

dotplot(gseahallmarks,showCategory=5,split=".sign")+facet_grid(~.sign)

library(export)

graph2ppt(file= "Results_1G.ppt", width=7, height=5)

####

#### Results 2A

####

#### Differential genes in TCGA

####

rm(list = ls())

load("TCGA_all_Diff_gene.Rdata")

rt <- read.table("inflammatory_response_gene.txt")

gene <- rt$V1

EMT <- all_Diff_gene[gene,]

IR <- na.omit(EMT)

IR <- IR[(IR$adj.P.Val < 0.05 & abs(IR$logFC) > 0.5),]

save(IR, file = "DEGs.Rdata")

####

#### Prognostic_genes in TCGA

####

rm(list = ls())

load("TCGA_LUAD_59Normal_513Tumor.Rdata")

test <- exprSet1[1:50,1:50]

library(dplyr)

library(tibble)

exprSet1 <- exprSet1 %>%

dplyr::filter(sample == "Tumor")

exprSet1 <- exprSet1[,-1]

exprSet1 <- exprSet1 %>%

rownames_to_column("ID")

exprSet2 <- exprSet1 %>%

mutate(probe_id = substring(exprSet1$ID,1,12)) %>%

select(ID,probe_id,everything())

test2 <- exprSet2[,1:10]

####

clin <- data.table::fread("Survival_SupplementalTable_S1_20171025_xena_sp",data.table = F)

colnames(clin)[3] <-"type"

colnames(clin)

table(clin$type)

TCGA_LUAD_survial <- clin %>%

filter(type == "LUAD")

LUAD_Clinical <- TCGA_LUAD_survial[,c(1,2,26,27)]

metadata1 <- data.frame(LUAD_Clinical$sample)

nrow(metadata1)

for (i in 1:nrow(metadata1)) {

num <- as.numeric(substring(metadata1[i,1],14,15))

if (num %in% seq(1,9)) {

metadata1[i,2] <- "Tumor"

}

if (num %in% seq(10,29)) {

metadata1[i,2] <- "Normal"

}

}

names(metadata1) <- c("sample","sample1")

LUAD_Clinical1 <- LUAD_Clinical %>%

inner_join(metadata1,by="sample") %>%

filter(sample1=="Tumor")

length(unique(LUAD_Clinical1$`_PATIENT`))

LUAD_Clinical2 <- LUAD_Clinical1[-c(133,147),]

length(unique(LUAD_Clinical2$`_PATIENT`))

LUAD_Clinical2 <- LUAD_Clinical2[,-c(1,5)]

colnames(LUAD_Clinical2) <- c("probe_id","fustat","futime")

####

exprSet3 <- exprSet2 %>%

inner_join(LUAD_Clinical2,by="probe_id") %>%

select(ID,probe_id,futime,fustat,everything())

exprSet3 <- exprSet3[,-2]

rownames(exprSet3) <- exprSet3$ID

exprSet3 <- exprSet3[,-1]

exprSet3 <- exprSet3 %>%

filter(futime > "0")

exprSet3 <- na.omit(exprSet3)

save(exprSet3,file = "TCGA_LUAD_500tumor_OS.Rdata")

####

test <- exprSet3[,1:10]

rt <- exprSet3

rt$futime <- rt$futime/30

IR_gene <- read.table("inflammatory_response_gene.txt")

same <- intersect(colnames(rt), IR_gene$V1)

IR <- rt[,same]

rt1 <- cbind(rt[,c(1:2)],IR)

rt <- rt1

genes <- colnames(rt)[-c(1:2)][1:198]

res2 <- data.frame()

library(survival)

####

#### Obtain survival P.value by KM method

####

for (i in 1:length(genes)) {

print(i)

surv =as.formula(paste('Surv(futime, fustat)~', "group"))

group = ifelse(rt[,genes[i]] > median(rt[,genes[i]]),"high","low")

if(length(table(group))==1) next

data = cbind(rt[,1:2],group)

x = survdiff(surv, data = data)

pValue=1-pchisq(x$chisq,df=1)

res2[i,1] = genes[i]

res2[i,2] = pValue

}

names(res2) <- c("ID","pValue_log")

####

#### Obtain survival P.value by cox method

####

genes <- colnames(rt)[-c(1:2)][1:198]

res <- data.frame()

for (i in 1:length(genes)) {

print(i)

surv = as.formula(paste('Surv(futime, fustat)~', genes[i]))

x = coxph(surv, data = rt)

x = summary(x)

p.value=signif(x$wald["pvalue"], digits=2)

HR =signif(x$coef[2], digits=2)

HR.confint.lower = signif(x$conf.int[,"lower .95"], 2)

HR.confint.upper = signif(x$conf.int[,"upper .95"], 2)

CI <- paste0("(", HR.confint.lower, "-", HR.confint.upper, ")")

res[i,1] = genes[i]

res[i,2] = HR

res[i,3] = CI

res[i,4] = p.value

}

names(res) <- c("ID","HR","95% CI","HR_p.value")

sur <- res %>%

inner_join(res2, by = "ID")

sur_p <- sur[(sur$HR_p.value < 0.05 & sur$pValue_log < 0.05),]

save(sur_p, file = "Prognostic_genes.Rdata")

####

#### venn

####

rm(list = ls())

library(VennDiagram)

library(ggplot2)

load("Prognostic_genes.Rdata")

load("DEGs.Rdata")

venn <- venn.diagram(list(DEGs = na.omit(rownames(IR)), Prognostic_genes = na.omit(sur_p$ID)),

filename = NULL, fill = c("darkorchid1","green"),

col = "black", alpha = 0.8, cat.cex =1.5, rotation.degree = 0)

grid.draw(venn)

library(export)

graph2ppt(file= "Results_2A.ppt", width=3, height=3)

####

#### Results 2B

####

rm(list = ls())

load("TCGA_LUAD_59Normal_513Tumor.Rdata")

sample <- exprSet1[,c(1,2)]

gene_14 <- read.table("14_venn_gene.txt")

gene <- gene_14$V1

exp <- exprSet1[,gene]

exprSet <- cbind(sample,exp)

exprSet <- exprSet[,-2]

save(exprSet,file = "14_gene_exp.Rdata")

####

Type <- exprSet$sample

names(Type) <- rownames(exprSet)

Type=as.data.frame(Type)

data <- as.matrix(t(exprSet[,-1]))

####

library(limma)

library(pheatmap)

library(reshape2)

library(ggpubr)

pheatmap(data,

annotation=Type,

color = colorRampPalette(c(rep("forestgreen",5), "white", rep("red",5)))(100),

cluster_cols =F,

cluster_rows =F,

scale="row",

show_colnames=F,

show_rownames=T,

fontsize=6,

fontsize_row=7,

fontsize_col=6)

library(export)

graph2ppt(file="Results_2B.ppt",width = 5, height = 3)

####

#### Results 2C

####

rm(list = ls())

library(igraph)

library(reshape2)

load("14_gene_exp.Rdata")

exp <- exprSet[c(exprSet$sample %in% "Tumor"),]

exp <- exp[,-1]

rt <- as.data.frame(t(exp))

geneRT <- read.table("14_venn_gene.txt",header=F,sep="\t",check.names=F)

data=t(rt[as.vector(geneRT[,1]),])

cordata=cor(data)

mydata = cordata

upper = upper.tri(mydata)

mydata[upper] = NA

df = data.frame(gene=rownames(mydata),mydata)

dfmeltdata = melt(df,id="gene")

dfmeltdata = dfmeltdata[!is.na(dfmeltdata$value),]

dfmeltdata = dfmeltdata[dfmeltdata$gene!=dfmeltdata$variable,]

cutoff = 0.3

dfmeltdata = dfmeltdata[abs(dfmeltdata$value)>cutoff,]

corweight = dfmeltdata$value

weight = corweight+abs(min(corweight))+5

d = data.frame(p1=dfmeltdata$gene,p2=dfmeltdata$variable,weight=dfmeltdata$value)

g = graph.data.frame(dfmeltdata,directed = FALSE)

E(g)$color = ifelse(corweight>0,rgb(254/255,67/255,101/255,abs(corweight)),rgb(0/255,0/255,255/255,abs(corweight)))

V(g)$size = 8

V(g)$shape = "circle"

V(g)$lable.cex = 1.2

V(g)$color = "white"

E(g)$weight = weight

layout(matrix(c(1,1,1,0,2,0),byrow=T,nc=3),height=c(6,1),width=c(3,4,3))

par(mar=c(1.5,2,2,2))

vertex.frame.color = NA

plot(g,layout=layout_nicely,vertex.label.cex=V(g)$lable.cex,edge.width = E(g)$weight,edge.arrow.size=0,vertex.label.color="black",vertex.frame.color=vertex.frame.color,edge.color=E(g)$color,vertex.label.cex=V(g)$lable.cex,vertex.label.font=2,vertex.size=V(g)$size,edge.curved=0.4)

color_legend = c(rgb(254/255,67/255,101/255,seq(1,0,by=-0.01)),rgb(0/255,0/255,255/255,seq(0,1,by=0.01)))

par(mar=c(2,2,1,2),xpd = T,cex.axis=1.6,las=1)

barplot(rep(1,length(color_legend)),border = NA, space = 0,ylab="",xlab="",xlim=c(1,length(color_legend)),horiz=FALSE,

axes = F, col=color_legend,main="")

axis(3,at=seq(1,length(color_legend),length=5),c(1,0.5,0,-0.5,-1),tick=FALSE)

library(export)

graph2ppt(file="Results_2C.ppt",width = 12, height = 7)

####

#### Results 2D

####

rm(list = ls())

load("TCGA_LUAD_500tumor_OS.Rdata")

venn_gene <- read.table("14_venn_gene.txt")

gene <- venn_gene$V1

exp <- exprSet3[,gene]

sur <- exprSet3[,c(1,2)]

exprSet <- cbind(sur,exp)

library("survival")

library("survminer")

train <- exprSet

outTab=data.frame()

sigGenes=c("futime","fustat")

for (i in colnames(train[,3:ncol(train)])) {

cox <- coxph(Surv(futime,fustat) ~ train[,i],data = train)

coxSummary = summary(cox)

coxP=coxSummary$coefficients[,"Pr(>|z|)"]

outTab=rbind(outTab,

cbind(id=i,

HR=coxSummary$conf.int[,"exp(coef)"],

HR.95L=coxSummary$conf.int[,"lower .95"],

HR.95H=coxSummary$conf.int[,"upper .95"],

pvalue=coxSummary$coefficients[,"Pr(>|z|)"]))

}

write.table(outTab,file="unicox_HR.txt",sep="\t",row.names=F,quote=F)

####

bioForest=function(coxFile=null,forestFile=null,height=null,forestCol=null){

rt <- read.table(coxFile,header=T,sep="\t",row.names=1,check.names=F)

gene <- rownames(rt)

hr <- sprintf("%.3f",rt$"HR")

hrLow <- sprintf("%.3f",rt$"HR.95L")

hrHigh <- sprintf("%.3f",rt$"HR.95H")

Hazard.ratio <- paste0(hr,"(",hrLow,"-",hrHigh,")")

pVal <- ifelse(rt$pvalue<0.001, "<0.001", sprintf("%.3f", rt$pvalue))

####

n <- nrow(rt)

nRow <- n+1

ylim <- c(1,nRow)

layout(matrix(c(1,2),nc=2),width=c(3,2.5))

####

xlim = c(0,3)

par(mar=c(4,2.5,2,1))

plot(1,xlim=xlim,ylim=ylim,type="n",axes=F,xlab="",ylab="")

text.cex=0.8

text(0,n:1,gene,adj=0,cex=text.cex)

text(1.5-0.5*0.2,n:1,pVal,adj=1,cex=text.cex);text(1.5-0.5*0.2,n+1,'pvalue',cex=text.cex,adj=1)

text(3,n:1,Hazard.ratio,adj=1,cex=text.cex);text(3,n+1,'Hazard ratio',cex=text.cex,adj=1,)

####

par(mar=c(4,1,2,1),mgp=c(2,0.5,0))

xlim = c(0,max(as.numeric(hrLow),as.numeric(hrHigh)))

plot(1,xlim=xlim,ylim=ylim,type="n",axes=F,ylab="",xaxs="i",xlab="Hazard ratio")

arrows(as.numeric(hrLow),n:1,as.numeric(hrHigh),n:1,angle=90,code=3,length=0.05,col="darkblue",lwd=3)

abline(v=1,col="black",lty=2,lwd=2)

boxcolor = ifelse(as.numeric(hr) > 1, forestCol[1], forestCol[2])

points(as.numeric(hr), n:1, pch = 15, col = boxcolor, cex=1.6)

axis(1)

}

bioForest(coxFile="unicox_HR.txt",forestFile="uniForest.pdf", forestCol=c("brown1","forestgreen"))

library(export)

graph2ppt(file = "Results_2D.ppt",width=8, height=6)

####

#### prepare train cohort and 8 validation cohort

####

#### TCGA cohort was recognized as train cohort

####

rm(list = ls())

load("TCGA_LUAD_500tumor_OS.Rdata")

gene_14 <- read.table("14_venn_gene.txt")

TCGA_exp <- exprSet3[,colnames(exprSet3) %in% gene_14$V1]

TCGA_sur <- exprSet3[,c(1:2)]

train <- cbind(TCGA_sur,TCGA_exp)

save(train,file = "train.Rdata")

####

#### GSE30219 was recognized as validation cohort 1

####

rm(list = ls())

load("GSE30219_85_LUAD.Rdata")

gene_14 <- read.table("14_venn_gene.txt")

GSE30219_exp <- exprSet4[,colnames(exprSet4) %in% gene_14$V1]

GSE30219_sur <- exprSet4[,c(1:2)]

test_1 <- cbind(GSE30219_sur,GSE30219_exp)

save(test_1,file = "test_1.Rdata")

####

#### GSE31210 was recognized as validation cohort 2

####

rm(list = ls())

load("GSE31210_226_LUAD.Rdata")

gene_14 <- read.table("14_venn_gene.txt")

GSE31210_exp <- exprSet4[,colnames(exprSet4) %in% gene_14$V1]

GSE31210_sur <- exprSet4[,c(1:2)]

test_2 <- cbind(GSE31210_sur,GSE31210_exp)

save(test_2,file = "test_2.Rdata")

####

#### GSE72094 was recognized as validation cohort 3

####

rm(list = ls())

load("GSE72094_398_LUAD.Rdata")

gene_14 <- read.table("14_venn_gene.txt")

GSE72094_exp <- exprSet4[,colnames(exprSet4) %in% gene_14$V1]

GSE72094_sur <- exprSet4[,c(1:2)]

test_3 <- cbind(GSE72094_sur,GSE72094_exp)

save(test_3,file = "test_3.Rdata")

####

#### GSE68465 was recognized as validation cohort 4

####

rm(list = ls())

load("GSE68465_442_LUAD.Rdata")

gene_14 <- read.table("14_venn_gene.txt")

GSE68465_exp <- exprSet4[,colnames(exprSet4) %in% gene_14$V1]

GSE68465_sur <- exprSet4[,c(1:2)]

test_4 <- cbind(GSE68465_sur,GSE68465_exp)

save(test_4,file = "test_4.Rdata")

####

#### GSE41271 was recognized as validation cohort 5

####

rm(list = ls())

load("GSE41271_182_LUAD.Rdata")

gene_14 <- read.table("14_venn_gene.txt")

GSE41271_exp <- exprSet4[,colnames(exprSet4) %in% gene_14$V1]

GSE41271_sur <- exprSet4[,c(1:2)]

test_5 <- cbind(GSE41271_sur,GSE41271_exp)

save(test_5,file = "test_5.Rdata")

####

#### GSE42127 was recognized as validation cohort 6

####

rm(list = ls())

load("GSE42127_133_LUAD.Rdata")

gene_14 <- read.table("14_venn_gene.txt")

GSE42127_exp <- exprSet4[,colnames(exprSet4) %in% gene_14$V1]

GSE42127_sur <- exprSet4[,c(1:2)]

test_6 <- cbind(GSE42127_sur,GSE42127_exp)

save(test_6,file = "test_6.Rdata")

####

#### GSE50081 was recognized as validation cohort 7

####

rm(list = ls())

load("GSE50081_127_LUAD.Rdata")

gene_14 <- read.table("14_venn_gene.txt")

GSE50081_exp <- exprSet4[,colnames(exprSet4) %in% gene_14$V1]

GSE50081_sur <- exprSet4[,c(1:2)]

test_7 <- cbind(GSE50081_sur,GSE50081_exp)

save(test_7,file = "test_7.Rdata")

####

#### GSE26939 was recognized as validation cohort 8

####

rm(list = ls())

load("GSE26939_115_LUAD.Rdata")

gene_14 <- read.table("14_venn_gene.txt")

GSE26939_exp <- exprSet4[,colnames(exprSet4) %in% gene_14$V1]

GSE26939_sur <- exprSet4[,c(1:2)]

test_8 <- cbind(GSE26939_sur,GSE26939_exp)

save(test_8,file = "test_8.Rdata")

####

#### lasso regression analyses

####

rm(list = ls())

library(glmnet)

library(survival)

load("train.Rdata")

trainLasso <- train

trainLasso$futime <- trainLasso$futime/365

x = as.matrix(trainLasso[,c(3:ncol(trainLasso))])

y = data.matrix(Surv(trainLasso$futime,trainLasso$fustat))

fit <- glmnet(x,y,family = "cox",maxit = 1000)

plot(fit,xvar = "lambda",label = TRUE)

library(export)

graph2ppt(file= "Results_2F.ppt",width=6,height=6)

####

cvfit <- cv.glmnet(x,y,family = "cox",maxit = 1000)

plot(cvfit)

abline(v=log(c(cvfit$lambda.min,cvfit$lambda.1se)),lty="dashed")

graph2ppt(file= "Results_2E.ppt",width=6,height=6)

####

coef <- coef(fit, s = cvfit$lambda.min)

index <- which(coef != 0)

actCoef <- coef[index]

lassoGene=row.names(coef)[index]

lassoGene=c("futime","fustat",lassoGene)

lassoSigExp=trainLasso[,lassoGene]

lassoSigExp=cbind(id=row.names(lassoSigExp),lassoSigExp)

write.table(lassoSigExp,file="lassoSigExp.txt",sep="\t",quote=F,row.names = F)

####

#### multivariate Cox regression analyses

####

load("train.Rdata")

load("test_1.Rdata")

load("test_2.Rdata")

load("test_3.Rdata")

load("test_4.Rdata")

load("test_5.Rdata")

load("test_6.Rdata")

load("test_7.Rdata")

load("test_8.Rdata")

train$futime <- train$futime/365

test_1$futime <- test_1$futime/365

test_2$futime <- test_2$futime/365

test_3$futime <- test_3$futime/365

test_4$futime <- test_4$futime/365

test_5$futime <- test_5$futime/365

test_6$futime <- test_6$futime/365

test_7$futime <- test_7$futime/365

test_8$futime <- test_8$futime*30

test_8$futime <- test_8$futime/365

####

train <- train[,lassoGene]

multiCox=coxph(Surv(futime, fustat) ~ ., data = train)

multiCox=step(multiCox,direction = "both")

multiCoxSum=summary(multiCox)

####

outMultiTab=data.frame()

outMultiTab=cbind(

coef=multiCoxSum$coefficients[,"coef"],

HR=multiCoxSum$conf.int[,"exp(coef)"],

HR.95L=multiCoxSum$conf.int[,"lower .95"],

HR.95H=multiCoxSum$conf.int[,"upper .95"],

pvalue=multiCoxSum$coefficients[,"Pr(>|z|)"])

outMultiTab=cbind(id=row.names(outMultiTab),outMultiTab)

outMultiTab=gsub("`","",outMultiTab)

write.table(outMultiTab,file="multiCox.xls",sep="\t",row.names=F,quote=F)

####

multiGene <- c("futime","fustat",rownames(outMultiTab))

test_1 <- test_1[,multiGene]

test_2 <- test_2[,multiGene]

test_3 <- test_3[,multiGene]

test_4 <- test_4[,multiGene]

test_5 <- test_5[,multiGene]

test_6 <- test_6[,multiGene]

test_7 <- test_7[,multiGene]

test_8 <- test_8[,multiGene]

####

#### train

####

riskScore = predict(multiCox,type="risk",newdata=train)

coxGene = rownames(multiCoxSum$coefficients)

coxGene = gsub("`","",coxGene)

outCol = c("futime","fustat",coxGene)

trainRiskOut = cbind(id=rownames(cbind(train[,outCol],riskScore,risk)),cbind(train[,outCol],riskScore))

####

#### test 1

####

riskScoreTest_1 = predict(multiCox,type="risk",newdata=test_1)

TestRiskOut_1 = cbind(id=rownames(cbind(test_1[,outCol],riskScoreTest_1,riskTest_1)),cbind(test_1[,outCol],riskScoreTest_1))

####

#### test 2

####

riskScoreTest_2 = predict(multiCox,type="risk",newdata=test_2)

TestRiskOut_2 = cbind(id=rownames(cbind(test_2[,outCol],riskScoreTest_2,riskTest_2)),cbind(test_2[,outCol],riskScoreTest_2))

####

#### test 3

####

riskScoreTest_3 = predict(multiCox,type="risk",newdata=test_3)

TestRiskOut_3 = cbind(id=rownames(cbind(test_3[,outCol],riskScoreTest_3,riskTest_3)),cbind(test_3[,outCol],riskScoreTest_3))

####

#### test 4

####

riskScoreTest_4 = predict(multiCox,type="risk",newdata=test_4)

TestRiskOut_4 = cbind(id=rownames(cbind(test_4[,outCol],riskScoreTest_4,riskTest_4)),cbind(test_4[,outCol],riskScoreTest_4))

####

#### test 5

####

riskScoreTest_5 = predict(multiCox,type="risk",newdata=test_5)

TestRiskOut_5 = cbind(id=rownames(cbind(test_5[,outCol],riskScoreTest_5,riskTest_5)),cbind(test_5[,outCol],riskScoreTest_5))

####

#### test 6

####

riskScoreTest_6 = predict(multiCox,type="risk",newdata=test_6)

TestRiskOut_6 = cbind(id=rownames(cbind(test_6[,outCol],riskScoreTest_6,riskTest_6)),cbind(test_6[,outCol],riskScoreTest_6))

####

#### test 7

####

riskScoreTest_7 = predict(multiCox,type="risk",newdata=test_7)

TestRiskOut_7 = cbind(id=rownames(cbind(test_7[,outCol],riskScoreTest_7,riskTest_7)),cbind(test_7[,outCol],riskScoreTest_7))

####

#### test 8

####

riskScoreTest_8 = predict(multiCox,type="risk",newdata=test_8)

TestRiskOut_8 = cbind(id=rownames(cbind(test_8[,outCol],riskScoreTest_8,riskTest_8)),cbind(test_8[,outCol],riskScoreTest_8))

####

write.table(trainRiskOut,file="train_risk.txt",sep="\t",quote=F,row.names = F)

write.table(TestRiskOut_1,file="test_1_risk.txt",sep="\t",quote=F,row.names = F)

write.table(TestRiskOut_2,file="test_2_risk.txt",sep="\t",quote=F,row.names = F)

write.table(TestRiskOut_3,file="test_3_risk.txt",sep="\t",quote=F,row.names = F)

write.table(TestRiskOut_4,file="test_4_risk.txt",sep="\t",quote=F,row.names = F)

write.table(TestRiskOut_5,file="test_5_risk.txt",sep="\t",quote=F,row.names = F)

write.table(TestRiskOut_6,file="test_6_risk.txt",sep="\t",quote=F,row.names = F)

write.table(TestRiskOut_7,file="test_7_risk.txt",sep="\t",quote=F,row.names = F)

write.table(TestRiskOut_8,file="test_8_risk.txt",sep="\t",quote=F,row.names = F)

####

#### train

####

rm(list = ls())

library(survival)

library(survminer)

data = read.table("train_risk.txt",header=T,sep="\t",check.names=F)

rt <- data[,c(2,3,11)]

colnames(rt)[3] <- c("riskScore")

rownames(rt) <- data$id

source("survivalROC_NEW.R")

cutoffROC <- function(t,rt){

rt=rt

ROC_rt <- survivalROC_NEW(Stime=rt[,1], status=rt[,2], marker = rt[,3],

predict.time =t, method="KM")

youdenindex=max(ROC_rt$sensitivity+ROC_rt$specificity-1)

index = which(ROC_rt$sensitivity+ROC_rt$specificity-1==youdenindex)+1

result=c(ROC_rt$cut.values[index],ROC_rt$sensitivity[index-1],ROC_rt$specificity[index-1])

}

####

t <- 4

####

(res.cut <- cutoffROC(t,rt))

cutoff <- res.cut[1]

save(cutoff,file = "cutoff_train.Rdata")

####

gene <- "riskScore"

rt <- rt[,c("futime","fustat",gene)]

rt$risk <- ifelse(rt$riskScore > cutoff,"high","low")

table(rt$risk)

####

my.surv <- Surv(rt$futime, rt$fustat)

group <- rt$risk

survival_dat <- data.frame(group = group)

fit <- survfit(my.surv ~ group)

summary(fit)$table

sur_high <- round(summary(fit)$table[1,7],3)

sur_low <- round(summary(fit)$table[2,7],3)

median <- c(sur_high,sur_low)

if(T){

group <- factor(group, levels = c("low", "high"))

data.survdiff <- survdiff(my.surv ~ group)

p.val = 1 - pchisq(data.survdiff$chisq, length(data.survdiff$n) - 1)

x = summary(coxph(Surv(futime, fustat)~riskScore, data = rt))

HR = signif(x$coef[2], digits=3)

up95 = signif(x$conf.int[,"upper .95"],3)

low95 = signif(x$conf.int[,"lower .95"], 3)

HR <- paste("HR = ", round(HR,3), sep = "")

CI <- paste(paste(round(low95,3), round(up95,3), sep = " - "), sep = "")

rt <- rt[order(rt[,"riskScore"],decreasing = T),]

ggsurvplot(fit, data = survival_dat ,

ggtheme = theme_bw(),

conf.int = T,

conf.int.style = "ribbon",

censor = T,

break.time.by = 2,

surv.median.line = "hv",

palette = c("darkorange","deepskyblue"),

ncensor.plot = FALSE,

font.legend = 12,

pval = paste(paste("pvalue =", p.val), paste(HR,"(",CI,")"),

paste("Median OS = ",median[1]," vs ",median[2]),sep = "\n"))

}

library(export)

graph2ppt(file= "train_sur.ppt",width=7,height=6)

####

library(survival)

library(survminer)

library(timeROC)

library(survivalROC)

rocCol=c("red","blue","green","yellow")

aucText=c()

par(oma=c(0.5,1,0,1),font.lab=1.5,font.axis=1.5)

train_ROC <- rt

####

roc=survivalROC(Stime=train_ROC$futime, status=train_ROC$fustat, marker = train_ROC$riskScore, predict.time =1, method="KM")

plot(roc$FP, roc$TP, type="l", xlim=c(0,1), ylim=c(0,1),col=rocCol[1],

xlab="False positive rate", ylab="True positive rate",main = "1",

lwd = 2, cex.main=1.2, cex.lab=1.2, cex.axis=1.2, font=1.2)

aucText=c(aucText,paste0("AUC at 1 years: ",sprintf("%.3f",roc$AUC)))

abline(0,1)

roc$AUC

####

roc=survivalROC(Stime=train_ROC$futime, status=train_ROC$fustat, marker = train_ROC$riskScore, predict.time =2, method="KM")

aucText=c(aucText,paste0("AUC at 2 years: ",sprintf("%.3f",roc$AUC)))

lines(roc$FP, roc$TP, type="l", xlim=c(0,1), ylim=c(0,1),col=rocCol[2],lwd = 2)

####

roc=survivalROC(Stime=train_ROC$futime, status=train_ROC$fustat, marker = train_ROC$riskScore, predict.time =3, method="KM")

aucText=c(aucText,paste0("AUC at 3 years: ",sprintf("%.3f",roc$AUC)))

lines(roc$FP, roc$TP, type="l", xlim=c(0,1), ylim=c(0,1),col=rocCol[3],lwd = 2)

####

roc=survivalROC(Stime=train_ROC$futime, status=train_ROC$fustat, marker = train_ROC$riskScore, predict.time =4, method="KM")

aucText=c(aucText,paste0("AUC at 4 years: ",sprintf("%.3f",roc$AUC)))

lines(roc$FP, roc$TP, type="l", xlim=c(0,1), ylim=c(0,1),col=rocCol[4],lwd = 2)

legend("bottomright", aucText,lwd=2,bty="n",col=rocCol)

library(export)

graph2ppt(file= paste0("train_auc.ppt"),width=5,height=5)

####

#### risk

####

rt=rt[order(rt$riskScore),]

riskClass=rt[,"risk"]

lowLength=length(riskClass[riskClass=="low"])

highLength=length(riskClass[riskClass=="high"])

line=rt[,"riskScore"]

line[line>10]=10

plot(line,

type="p",

pch=20,

xlab="Patients (increasing risk socre)",

ylab="Risk score",

col=c(rep("green",lowLength),

rep("red",highLength)))

abline(h=cutoff,v=lowLength,lty=2)

legend("topleft", c("High risk", "low Risk"),bty="n",pch=19,col=c("red","green"),cex=1.2)

library(export)

graph2ppt(file= "train_risk_1.ppt",width=6,height=3.5)

####

color=as.vector(rt$fustat)

color[color==1]="red"

color[color==0]="green"

plot(rt$futime,

pch=19,

xlab="Patients (increasing risk socre)",

ylab="Survival time (years)",

col=color)

legend("topleft", c("Dead", "Alive"),bty="n",pch=19,col=c("red","green"),cex=1.2)

abline(v=lowLength,lty=2)

library(export)

graph2ppt(file= "train_risk_2.ppt",width=6,height=3.5)

####

#### HR

####

library("survival")

library("survminer")

gene <- colnames(rt)[3]

library(tibble)

library(dplyr)

rt1 <- rt %>%

select(futime,fustat,gene)

outTab=data.frame()

cox=coxph(Surv(futime, fustat) ~ rt1[,3], data = rt1)

coxSummary=summary(cox)

outTab=rbind(outTab,

cbind(id="train",

HR=coxSummary$conf.int[,"exp(coef)"],

HR.95L=coxSummary$conf.int[,"lower .95"],

HR.95H=coxSummary$conf.int[,"upper .95"],

pvalue=coxSummary$coefficients[,"Pr(>|z|)"])

)

write.table(outTab, file= "train_cox.txt", sep="\t", row.names=F, quote=F)

####

#### test_1

####

rm(list = ls())

data=read.table("test_1_risk.txt",header=T,sep="\t",check.names=F)

rt <- data[,c(2,3,11)]

colnames(rt)[3] <- c("riskScore")

rownames(rt) <- data$id

source("survivalROC_NEW.R")

cutoffROC <- function(t,rt){

rt=rt

ROC_rt <- survivalROC_NEW(Stime=rt[,1], status=rt[,2], marker = rt[,3],

predict.time =t, method="KM")

youdenindex=max(ROC_rt$sensitivity+ROC_rt$specificity-1)

index = which(ROC_rt$sensitivity+ROC_rt$specificity-1==youdenindex)+1

result=c(ROC_rt$cut.values[index],ROC_rt$sensitivity[index-1],ROC_rt$specificity[index-1])

}

####

t <- 4

####

(res.cut <- cutoffROC(t,rt))

cutoff <- res.cut[1]

save(cutoff,file = "cutoff_test_1.Rdata")

####

gene <- "riskScore"

rt <- rt[,c("futime","fustat",gene)]

rt$risk <- ifelse(rt$riskScore > cutoff,"high","low")

table(rt$risk)

####

my.surv <- Surv(rt$futime, rt$fustat)

group <- rt$risk

survival_dat <- data.frame(group = group)

fit <- survfit(my.surv ~ group)

summary(fit)$table

sur_high <- round(summary(fit)$table[1,7],3)

sur_low <- round(summary(fit)$table[2,7],3)

median <- c(sur_high,sur_low)

if(T){

group <- factor(group, levels = c("low", "high"))

data.survdiff <- survdiff(my.surv ~ group)

p.val = 1 - pchisq(data.survdiff$chisq, length(data.survdiff$n) - 1)

x = summary(coxph(Surv(futime, fustat)~riskScore, data = rt))

HR = signif(x$coef[2], digits=3)

up95 = signif(x$conf.int[,"upper .95"],3)

low95 = signif(x$conf.int[,"lower .95"], 3)

HR <- paste("HR = ", round(HR,3), sep = "")

CI <- paste(paste(round(low95,3), round(up95,3), sep = " - "), sep = "")

rt <- rt[order(rt[,"riskScore"],decreasing = T),]

ggsurvplot(fit, data = survival_dat ,

ggtheme = theme_bw(),

conf.int = T,

conf.int.style = "ribbon",

censor = T,

break.time.by = 2,

surv.median.line = "hv",

palette = c("darkorange","deepskyblue"),

ncensor.plot = FALSE,

font.legend = 12,

pval = paste(paste("pvalue =", p.val), paste(HR,"(",CI,")"),

paste("Median OS = ",median[1]," vs ",median[2]),sep = "\n"))

}

library(export)

graph2ppt(file= "test_1_sur.ppt",width=7,height=6)

####

library(survival)

library(survminer)

library(timeROC)

library(survivalROC)

rocCol=c("red","blue","green","yellow")

aucText=c()

par(oma=c(0.5,1,0,1),font.lab=1.5,font.axis=1.5)

train_ROC <- rt

####

roc=survivalROC(Stime=train_ROC$futime, status=train_ROC$fustat, marker = train_ROC$riskScore, predict.time =1, method="KM")

plot(roc$FP, roc$TP, type="l", xlim=c(0,1), ylim=c(0,1),col=rocCol[1],

xlab="False positive rate", ylab="True positive rate",main = "1",

lwd = 2, cex.main=1.2, cex.lab=1.2, cex.axis=1.2, font=1.2)

aucText=c(aucText,paste0("AUC at 1 years: ",sprintf("%.3f",roc$AUC)))

abline(0,1)

roc$AUC

####

roc=survivalROC(Stime=train_ROC$futime, status=train_ROC$fustat, marker = train_ROC$riskScore, predict.time =2, method="KM")

aucText=c(aucText,paste0("AUC at 2 years: ",sprintf("%.3f",roc$AUC)))

lines(roc$FP, roc$TP, type="l", xlim=c(0,1), ylim=c(0,1),col=rocCol[2],lwd = 2)

####

roc=survivalROC(Stime=train_ROC$futime, status=train_ROC$fustat, marker = train_ROC$riskScore, predict.time =3, method="KM")

aucText=c(aucText,paste0("AUC at 3 years: ",sprintf("%.3f",roc$AUC)))

lines(roc$FP, roc$TP, type="l", xlim=c(0,1), ylim=c(0,1),col=rocCol[3],lwd = 2)

####

roc=survivalROC(Stime=train_ROC$futime, status=train_ROC$fustat, marker = train_ROC$riskScore, predict.time =4, method="KM")

aucText=c(aucText,paste0("AUC at 4 years: ",sprintf("%.3f",roc$AUC)))

lines(roc$FP, roc$TP, type="l", xlim=c(0,1), ylim=c(0,1),col=rocCol[4],lwd = 2)

legend("bottomright", aucText,lwd=2,bty="n",col=rocCol)

library(export)

graph2ppt(file= paste0("test_1_auc.ppt"),width=5,height=5)

####

#### risk

####

rt=rt[order(rt$riskScore),]

riskClass=rt[,"risk"]

lowLength=length(riskClass[riskClass=="low"])

highLength=length(riskClass[riskClass=="high"])

line=rt[,"riskScore"]

line[line>10]=10

plot(line,

type="p",

pch=20,

xlab="Patients (increasing risk socre)",

ylab="Risk score",

col=c(rep("green",lowLength),

rep("red",highLength)))

abline(h=cutoff,v=lowLength,lty=2)

legend("topleft", c("High risk", "low Risk"),bty="n",pch=19,col=c("red","green"),cex=1.2)

library(export)

graph2ppt(file= "test_1_risk_1.ppt",width=6,height=3.5)

####

color=as.vector(rt$fustat)

color[color==1]="red"

color[color==0]="green"

plot(rt$futime,

pch=19,

xlab="Patients (increasing risk socre)",

ylab="Survival time (years)",

col=color)

legend("topleft", c("Dead", "Alive"),bty="n",pch=19,col=c("red","green"),cex=1.2)

abline(v=lowLength,lty=2)

library(export)

graph2ppt(file= "test_1_risk_2.ppt",width=6,height=3.5)

####

#### HR

####

library("survival")

library("survminer")

gene <- colnames(rt)[3]

library(tibble)

library(dplyr)

rt1 <- rt %>%

select(futime,fustat,gene)

outTab=data.frame()

cox=coxph(Surv(futime, fustat) ~ rt1[,3], data = rt1)

coxSummary=summary(cox)

outTab=rbind(outTab,

cbind(id="test 1",

HR=coxSummary$conf.int[,"exp(coef)"],

HR.95L=coxSummary$conf.int[,"lower .95"],

HR.95H=coxSummary$conf.int[,"upper .95"],

pvalue=coxSummary$coefficients[,"Pr(>|z|)"])

)

write.table(outTab, file= "test_1_cox.txt", sep="\t", row.names=F, quote=F)

####

#### test_2

####

rm(list = ls())

data=read.table("test_2_risk.txt",header=T,sep="\t",check.names=F)

rt <- data[,c(2,3,11)]

colnames(rt)[3] <- c("riskScore")

rownames(rt) <- data$id

source("survivalROC_NEW.R")

cutoffROC <- function(t,rt){

rt=rt

ROC_rt <- survivalROC_NEW(Stime=rt[,1], status=rt[,2], marker = rt[,3],

predict.time =t, method="KM")

youdenindex=max(ROC_rt$sensitivity+ROC_rt$specificity-1)

index = which(ROC_rt$sensitivity+ROC_rt$specificity-1==youdenindex)+1

result=c(ROC_rt$cut.values[index],ROC_rt$sensitivity[index-1],ROC_rt$specificity[index-1])

}

####

t <- 4

####

(res.cut <- cutoffROC(t,rt))

cutoff <- res.cut[1]

save(cutoff,file = "cutoff_test_2.Rdata")

####

gene <- "riskScore"

rt <- rt[,c("futime","fustat",gene)]

rt$risk <- ifelse(rt$riskScore > cutoff,"high","low")

table(rt$risk)

####

my.surv <- Surv(rt$futime, rt$fustat)

group <- rt$risk

survival_dat <- data.frame(group = group)

fit <- survfit(my.surv ~ group)

summary(fit)$table

sur_high <- round(summary(fit)$table[1,7],3)

sur_low <- round(summary(fit)$table[2,7],3)

median <- c(sur_high,sur_low)

if(T){

group <- factor(group, levels = c("low", "high"))

data.survdiff <- survdiff(my.surv ~ group)

p.val = 1 - pchisq(data.survdiff$chisq, length(data.survdiff$n) - 1)

x = summary(coxph(Surv(futime, fustat)~riskScore, data = rt))

HR = signif(x$coef[2], digits=3)

up95 = signif(x$conf.int[,"upper .95"],3)

low95 = signif(x$conf.int[,"lower .95"], 3)

HR <- paste("HR = ", round(HR,3), sep = "")

CI <- paste(paste(round(low95,3), round(up95,3), sep = " - "), sep = "")

rt <- rt[order(rt[,"riskScore"],decreasing = T),]

ggsurvplot(fit, data = survival_dat ,

ggtheme = theme_bw(),

conf.int = T,

conf.int.style = "ribbon",

censor = T,

break.time.by = 2,

surv.median.line = "hv",

palette = c("darkorange","deepskyblue"),

ncensor.plot = FALSE,

font.legend = 12,

pval = paste(paste("pvalue =", p.val), paste(HR,"(",CI,")"),

paste("Median OS = ",median[1]," vs ",median[2]),sep = "\n"))

}

library(export)

graph2ppt(file= "test_2_sur.ppt",width=7,height=6)

####

library(survival)

library(survminer)

library(timeROC)

library(survivalROC)

rocCol=c("red","blue","green","yellow")

aucText=c()

par(oma=c(0.5,1,0,1),font.lab=1.5,font.axis=1.5)

train_ROC <- rt

####

roc=survivalROC(Stime=train_ROC$futime, status=train_ROC$fustat, marker = train_ROC$riskScore, predict.time =1, method="KM")

plot(roc$FP, roc$TP, type="l", xlim=c(0,1), ylim=c(0,1),col=rocCol[1],

xlab="False positive rate", ylab="True positive rate",main = "1",

lwd = 2, cex.main=1.2, cex.lab=1.2, cex.axis=1.2, font=1.2)

aucText=c(aucText,paste0("AUC at 1 years: ",sprintf("%.3f",roc$AUC)))

abline(0,1)

roc$AUC

####

roc=survivalROC(Stime=train_ROC$futime, status=train_ROC$fustat, marker = train_ROC$riskScore, predict.time =2, method="KM")

aucText=c(aucText,paste0("AUC at 2 years: ",sprintf("%.3f",roc$AUC)))

lines(roc$FP, roc$TP, type="l", xlim=c(0,1), ylim=c(0,1),col=rocCol[2],lwd = 2)

####

roc=survivalROC(Stime=train_ROC$futime, status=train_ROC$fustat, marker = train_ROC$riskScore, predict.time =3, method="KM")

aucText=c(aucText,paste0("AUC at 3 years: ",sprintf("%.3f",roc$AUC)))

lines(roc$FP, roc$TP, type="l", xlim=c(0,1), ylim=c(0,1),col=rocCol[3],lwd = 2)

####

roc=survivalROC(Stime=train_ROC$futime, status=train_ROC$fustat, marker = train_ROC$riskScore, predict.time =4, method="KM")

aucText=c(aucText,paste0("AUC at 4 years: ",sprintf("%.3f",roc$AUC)))

lines(roc$FP, roc$TP, type="l", xlim=c(0,1), ylim=c(0,1),col=rocCol[4],lwd = 2)

legend("bottomright", aucText,lwd=2,bty="n",col=rocCol)

library(export)

graph2ppt(file= paste0("test_2_auc.ppt"),width=5,height=5)

####

#### risk

####

rt=rt[order(rt$riskScore),]

riskClass=rt[,"risk"]

lowLength=length(riskClass[riskClass=="low"])

highLength=length(riskClass[riskClass=="high"])

line=rt[,"riskScore"]

line[line>10]=10

plot(line,

type="p",

pch=20,

xlab="Patients (increasing risk socre)",

ylab="Risk score",

col=c(rep("green",lowLength),

rep("red",highLength)))

abline(h=cutoff,v=lowLength,lty=2)

legend("topleft", c("High risk", "low Risk"),bty="n",pch=19,col=c("red","green"),cex=1.2)

library(export)

graph2ppt(file= "test_2_risk_1.ppt",width=6,height=3.5)

####

color=as.vector(rt$fustat)

color[color==1]="red"

color[color==0]="green"

plot(rt$futime,

pch=19,

xlab="Patients (increasing risk socre)",

ylab="Survival time (years)",

col=color)

legend("topleft", c("Dead", "Alive"),bty="n",pch=19,col=c("red","green"),cex=1.2)

abline(v=lowLength,lty=2)

library(export)

graph2ppt(file= "test_2_risk_2.ppt",width=6,height=3.5)

####

#### HR

####

library("survival")

library("survminer")

gene <- colnames(rt)[3]

library(tibble)

library(dplyr)

rt1 <- rt %>%

select(futime,fustat,gene)

outTab=data.frame()

cox=coxph(Surv(futime, fustat) ~ rt1[,3], data = rt1)

coxSummary=summary(cox)

outTab=rbind(outTab,

cbind(id="test 2",

HR=coxSummary$conf.int[,"exp(coef)"],

HR.95L=coxSummary$conf.int[,"lower .95"],

HR.95H=coxSummary$conf.int[,"upper .95"],

pvalue=coxSummary$coefficients[,"Pr(>|z|)"])

)

write.table(outTab, file= "test_2_cox.txt", sep="\t", row.names=F, quote=F)

####

#### test_3

####

rm(list = ls())

data=read.table("test_3_risk.txt",header=T,sep="\t",check.names=F)

rt <- data[,c(2,3,11)]

colnames(rt)[3] <- c("riskScore")

rownames(rt) <- data$id

source("survivalROC_NEW.R")

cutoffROC <- function(t,rt){

rt=rt

ROC_rt <- survivalROC_NEW(Stime=rt[,1], status=rt[,2], marker = rt[,3],

predict.time =t, method="KM")

youdenindex=max(ROC_rt$sensitivity+ROC_rt$specificity-1)

index = which(ROC_rt$sensitivity+ROC_rt$specificity-1==youdenindex)+1

result=c(ROC_rt$cut.values[index],ROC_rt$sensitivity[index-1],ROC_rt$specificity[index-1])

}

####

t <- 4

####

(res.cut <- cutoffROC(t,rt))

cutoff <- res.cut[1]

save(cutoff,file = "cutoff_test_3.Rdata")

####

gene <- "riskScore"

rt <- rt[,c("futime","fustat",gene)]

rt$risk <- ifelse(rt$riskScore > cutoff,"high","low")

table(rt$risk)

####

my.surv <- Surv(rt$futime, rt$fustat)

group <- rt$risk

survival_dat <- data.frame(group = group)

fit <- survfit(my.surv ~ group)

summary(fit)$table

sur_high <- round(summary(fit)$table[1,7],3)

sur_low <- round(summary(fit)$table[2,7],3)

median <- c(sur_high,sur_low)

if(T){

group <- factor(group, levels = c("low", "high"))

data.survdiff <- survdiff(my.surv ~ group)

p.val = 1 - pchisq(data.survdiff$chisq, length(data.survdiff$n) - 1)

x = summary(coxph(Surv(futime, fustat)~riskScore, data = rt))

HR = signif(x$coef[2], digits=3)

up95 = signif(x$conf.int[,"upper .95"],3)

low95 = signif(x$conf.int[,"lower .95"], 3)

HR <- paste("HR = ", round(HR,3), sep = "")

CI <- paste(paste(round(low95,3), round(up95,3), sep = " - "), sep = "")

rt <- rt[order(rt[,"riskScore"],decreasing = T),]

ggsurvplot(fit, data = survival_dat ,

ggtheme = theme_bw(),

conf.int = T,

conf.int.style = "ribbon",

censor = T,

break.time.by = 2,

surv.median.line = "hv",

palette = c("darkorange","deepskyblue"),

ncensor.plot = FALSE,

font.legend = 12,

pval = paste(paste("pvalue =", p.val), paste(HR,"(",CI,")"),

paste("Median OS = ",median[1]," vs ",median[2]),sep = "\n"))

}

library(export)

graph2ppt(file= "test_3_sur.ppt",width=7,height=6)

####

#### HR

####

library("survival")

library("survminer")

gene <- colnames(rt)[3]

library(tibble)

library(dplyr)

rt1 <- rt %>%

select(futime,fustat,gene)

outTab=data.frame()

cox=coxph(Surv(futime, fustat) ~ rt1[,3], data = rt1)

coxSummary=summary(cox)

outTab=rbind(outTab,

cbind(id="test 3",

HR=coxSummary$conf.int[,"exp(coef)"],

HR.95L=coxSummary$conf.int[,"lower .95"],

HR.95H=coxSummary$conf.int[,"upper .95"],

pvalue=coxSummary$coefficients[,"Pr(>|z|)"])

)

write.table(outTab, file= "test_3_cox.txt", sep="\t", row.names=F, quote=F)

####

#### test_4

####

rm(list = ls())

data=read.table("test_4_risk.txt",header=T,sep="\t",check.names=F)

rt <- data[,c(2,3,11)]

colnames(rt)[3] <- c("riskScore")

rownames(rt) <- data$id

source("survivalROC_NEW.R")

cutoffROC <- function(t,rt){

rt=rt

ROC_rt <- survivalROC_NEW(Stime=rt[,1], status=rt[,2], marker = rt[,3],

predict.time =t, method="KM")

youdenindex=max(ROC_rt$sensitivity+ROC_rt$specificity-1)

index = which(ROC_rt$sensitivity+ROC_rt$specificity-1==youdenindex)+1

result=c(ROC_rt$cut.values[index],ROC_rt$sensitivity[index-1],ROC_rt$specificity[index-1])

}

####

t <- 4

####

(res.cut <- cutoffROC(t,rt))

cutoff <- res.cut[1]

save(cutoff,file = "cutoff_test_4.Rdata")

####

gene <- "riskScore"

rt <- rt[,c("futime","fustat",gene)]

rt$risk <- ifelse(rt$riskScore > cutoff,"high","low")

table(rt$risk)

####

my.surv <- Surv(rt$futime, rt$fustat)

group <- rt$risk

survival_dat <- data.frame(group = group)

fit <- survfit(my.surv ~ group)

summary(fit)$table

sur_high <- round(summary(fit)$table[1,7],3)

sur_low <- round(summary(fit)$table[2,7],3)

median <- c(sur_high,sur_low)

if(T){

group <- factor(group, levels = c("low", "high"))

data.survdiff <- survdiff(my.surv ~ group)

p.val = 1 - pchisq(data.survdiff$chisq, length(data.survdiff$n) - 1)

x = summary(coxph(Surv(futime, fustat)~riskScore, data = rt))

HR = signif(x$coef[2], digits=3)

up95 = signif(x$conf.int[,"upper .95"],3)

low95 = signif(x$conf.int[,"lower .95"], 3)

HR <- paste("HR = ", round(HR,3), sep = "")

CI <- paste(paste(round(low95,3), round(up95,3), sep = " - "), sep = "")

rt <- rt[order(rt[,"riskScore"],decreasing = T),]

ggsurvplot(fit, data = survival_dat ,

ggtheme = theme_bw(),

conf.int = T,

conf.int.style = "ribbon",

censor = T,

break.time.by = 2,

surv.median.line = "hv",

palette = c("darkorange","deepskyblue"),

ncensor.plot = FALSE,

font.legend = 12,

pval = paste(paste("pvalue =", p.val), paste(HR,"(",CI,")"),

paste("Median OS = ",median[1]," vs ",median[2]),sep = "\n"))

}

library(export)

graph2ppt(file= "test_4_sur.ppt",width=7,height=6)

####

#### HR

####

library("survival")

library("survminer")

gene <- colnames(rt)[3]

library(tibble)

library(dplyr)

rt1 <- rt %>%

select(futime,fustat,gene)

outTab=data.frame()

cox=coxph(Surv(futime, fustat) ~ rt1[,3], data = rt1)

coxSummary=summary(cox)

outTab=rbind(outTab,

cbind(id="test 4",

HR=coxSummary$conf.int[,"exp(coef)"],

HR.95L=coxSummary$conf.int[,"lower .95"],

HR.95H=coxSummary$conf.int[,"upper .95"],

pvalue=coxSummary$coefficients[,"Pr(>|z|)"])

)

write.table(outTab, file= "test_4_cox.txt", sep="\t", row.names=F, quote=F)

####

#### test_5

####

rm(list = ls())

data=read.table("test_5_risk.txt",header=T,sep="\t",check.names=F)

rt <- data[,c(2,3,11)]

colnames(rt)[3] <- c("riskScore")

rownames(rt) <- data$id

source("survivalROC_NEW.R")

cutoffROC <- function(t,rt){

rt=rt

ROC_rt <- survivalROC_NEW(Stime=rt[,1], status=rt[,2], marker = rt[,3],

predict.time =t, method="KM")

youdenindex=max(ROC_rt$sensitivity+ROC_rt$specificity-1)

index = which(ROC_rt$sensitivity+ROC_rt$specificity-1==youdenindex)+1

result=c(ROC_rt$cut.values[index],ROC_rt$sensitivity[index-1],ROC_rt$specificity[index-1])

}

####

t <- 4

####

(res.cut <- cutoffROC(t,rt))

cutoff <- res.cut[1]

save(cutoff,file = "cutoff_test_5.Rdata")

####

gene <- "riskScore"

rt <- rt[,c("futime","fustat",gene)]

rt$risk <- ifelse(rt$riskScore > cutoff,"high","low")

table(rt$risk)

####

my.surv <- Surv(rt$futime, rt$fustat)

group <- rt$risk

survival_dat <- data.frame(group = group)

fit <- survfit(my.surv ~ group)

summary(fit)$table

sur_high <- round(summary(fit)$table[1,7],3)

sur_low <- round(summary(fit)$table[2,7],3)

median <- c(sur_high,sur_low)

if(T){

group <- factor(group, levels = c("low", "high"))

data.survdiff <- survdiff(my.surv ~ group)

p.val = 1 - pchisq(data.survdiff$chisq, length(data.survdiff$n) - 1)

x = summary(coxph(Surv(futime, fustat)~riskScore, data = rt))

HR = signif(x$coef[2], digits=3)

up95 = signif(x$conf.int[,"upper .95"],3)

low95 = signif(x$conf.int[,"lower .95"], 3)

HR <- paste("HR = ", round(HR,3), sep = "")

CI <- paste(paste(round(low95,3), round(up95,3), sep = " - "), sep = "")

rt <- rt[order(rt[,"riskScore"],decreasing = T),]

ggsurvplot(fit, data = survival_dat ,

ggtheme = theme_bw(),

conf.int = T,

conf.int.style = "ribbon",

censor = T,

break.time.by = 2,

surv.median.line = "hv",

palette = c("darkorange","deepskyblue"),

ncensor.plot = FALSE,

font.legend = 12,

pval = paste(paste("pvalue =", p.val), paste(HR,"(",CI,")"),

paste("Median OS = ",median[1]," vs ",median[2]),sep = "\n"))

}

library(export)

graph2ppt(file= "test_5_sur.ppt",width=7,height=6)

####

#### HR

####

library("survival")

library("survminer")

gene <- colnames(rt)[3]

library(tibble)

library(dplyr)

rt1 <- rt %>%

select(futime,fustat,gene)

outTab=data.frame()

cox=coxph(Surv(futime, fustat) ~ rt1[,3], data = rt1)

coxSummary=summary(cox)

outTab=rbind(outTab,

cbind(id="test 5",

HR=coxSummary$conf.int[,"exp(coef)"],

HR.95L=coxSummary$conf.int[,"lower .95"],

HR.95H=coxSummary$conf.int[,"upper .95"],

pvalue=coxSummary$coefficients[,"Pr(>|z|)"])

)

write.table(outTab, file= "test_5_cox.txt", sep="\t", row.names=F, quote=F)

####

#### test_6

####

rm(list = ls())

data=read.table("test_6_risk.txt",header=T,sep="\t",check.names=F)

rt <- data[,c(2,3,11)]

colnames(rt)[3] <- c("riskScore")

rownames(rt) <- data$id

source("survivalROC_NEW.R")

cutoffROC <- function(t,rt){

rt=rt

ROC_rt <- survivalROC_NEW(Stime=rt[,1], status=rt[,2], marker = rt[,3],

predict.time =t, method="KM")

youdenindex=max(ROC_rt$sensitivity+ROC_rt$specificity-1)

index = which(ROC_rt$sensitivity+ROC_rt$specificity-1==youdenindex)+1

result=c(ROC_rt$cut.values[index],ROC_rt$sensitivity[index-1],ROC_rt$specificity[index-1])

}

####

t <- 4

####

(res.cut <- cutoffROC(t,rt))

cutoff <- res.cut[1]

save(cutoff,file = "cutoff_test_6.Rdata")

####

gene <- "riskScore"

rt <- rt[,c("futime","fustat",gene)]

rt$risk <- ifelse(rt$riskScore > cutoff,"high","low")

table(rt$risk)

####

my.surv <- Surv(rt$futime, rt$fustat)

group <- rt$risk

survival_dat <- data.frame(group = group)

fit <- survfit(my.surv ~ group)

summary(fit)$table

sur_high <- round(summary(fit)$table[1,7],3)

sur_low <- round(summary(fit)$table[2,7],3)

median <- c(sur_high,sur_low)

if(T){

group <- factor(group, levels = c("low", "high"))

data.survdiff <- survdiff(my.surv ~ group)

p.val = 1 - pchisq(data.survdiff$chisq, length(data.survdiff$n) - 1)

x = summary(coxph(Surv(futime, fustat)~riskScore, data = rt))

HR = signif(x$coef[2], digits=3)

up95 = signif(x$conf.int[,"upper .95"],3)

low95 = signif(x$conf.int[,"lower .95"], 3)

HR <- paste("HR = ", round(HR,3), sep = "")

CI <- paste(paste(round(low95,3), round(up95,3), sep = " - "), sep = "")

rt <- rt[order(rt[,"riskScore"],decreasing = T),]

ggsurvplot(fit, data = survival_dat ,

ggtheme = theme_bw(),

conf.int = T,

conf.int.style = "ribbon",

censor = T,

break.time.by = 2,

surv.median.line = "hv",

palette = c("darkorange","deepskyblue"),

ncensor.plot = FALSE,

font.legend = 12,

pval = paste(paste("pvalue =", p.val), paste(HR,"(",CI,")"),

paste("Median OS = ",median[1]," vs ",median[2]),sep = "\n"))

}

library(export)

graph2ppt(file= "test_6_sur.ppt",width=7,height=6)

####

#### HR

####

library("survival")

library("survminer")

gene <- colnames(rt)[3]

library(tibble)

library(dplyr)

rt1 <- rt %>%

select(futime,fustat,gene)

outTab=data.frame()

cox=coxph(Surv(futime, fustat) ~ rt1[,3], data = rt1)

coxSummary=summary(cox)

outTab=rbind(outTab,

cbind(id="test 6",

HR=coxSummary$conf.int[,"exp(coef)"],

HR.95L=coxSummary$conf.int[,"lower .95"],

HR.95H=coxSummary$conf.int[,"upper .95"],

pvalue=coxSummary$coefficients[,"Pr(>|z|)"])

)

write.table(outTab, file= "test_6_cox.txt", sep="\t", row.names=F, quote=F)

####

#### test_7

####

rm(list = ls())

data=read.table("test_7_risk.txt",header=T,sep="\t",check.names=F)

rt <- data[,c(2,3,11)]

colnames(rt)[3] <- c("riskScore")

rownames(rt) <- data$id

source("survivalROC_NEW.R")

cutoffROC <- function(t,rt){

rt=rt

ROC_rt <- survivalROC_NEW(Stime=rt[,1], status=rt[,2], marker = rt[,3],

predict.time =t, method="KM")

youdenindex=max(ROC_rt$sensitivity+ROC_rt$specificity-1)

index = which(ROC_rt$sensitivity+ROC_rt$specificity-1==youdenindex)+1

result=c(ROC_rt$cut.values[index],ROC_rt$sensitivity[index-1],ROC_rt$specificity[index-1])

}

####

t <- 4

####

(res.cut <- cutoffROC(t,rt))

cutoff <- res.cut[1]

save(cutoff,file = "cutoff_test_7.Rdata")

####

gene <- "riskScore"

rt <- rt[,c("futime","fustat",gene)]

rt$risk <- ifelse(rt$riskScore > cutoff,"high","low")

table(rt$risk)

####

my.surv <- Surv(rt$futime, rt$fustat)

group <- rt$risk

survival_dat <- data.frame(group = group)

fit <- survfit(my.surv ~ group)

summary(fit)$table

sur_high <- round(summary(fit)$table[1,7],3)

sur_low <- round(summary(fit)$table[2,7],3)

median <- c(sur_high,sur_low)

if(T){

group <- factor(group, levels = c("low", "high"))

data.survdiff <- survdiff(my.surv ~ group)

p.val = 1 - pchisq(data.survdiff$chisq, length(data.survdiff$n) - 1)

x = summary(coxph(Surv(futime, fustat)~riskScore, data = rt))

HR = signif(x$coef[2], digits=3)

up95 = signif(x$conf.int[,"upper .95"],3)

low95 = signif(x$conf.int[,"lower .95"], 3)

HR <- paste("HR = ", round(HR,3), sep = "")

CI <- paste(paste(round(low95,3), round(up95,3), sep = " - "), sep = "")

rt <- rt[order(rt[,"riskScore"],decreasing = T),]

ggsurvplot(fit, data = survival_dat ,

ggtheme = theme_bw(),

conf.int = T,

conf.int.style = "ribbon",

censor = T,

break.time.by = 2,

surv.median.line = "hv",

palette = c("darkorange","deepskyblue"),

ncensor.plot = FALSE,

font.legend = 12,

pval = paste(paste("pvalue =", p.val), paste(HR,"(",CI,")"),

paste("Median OS = ",median[1]," vs ",median[2]),sep = "\n"))

}

library(export)

graph2ppt(file= "test_7_sur.ppt",width=7,height=6)

####

#### HR

####

library("survival")

library("survminer")

gene <- colnames(rt)[3]

library(tibble)

library(dplyr)

rt1 <- rt %>%

select(futime,fustat,gene)

outTab=data.frame()

cox=coxph(Surv(futime, fustat) ~ rt1[,3], data = rt1)

coxSummary=summary(cox)

outTab=rbind(outTab,

cbind(id="test 7",

HR=coxSummary$conf.int[,"exp(coef)"],

HR.95L=coxSummary$conf.int[,"lower .95"],

HR.95H=coxSummary$conf.int[,"upper .95"],

pvalue=coxSummary$coefficients[,"Pr(>|z|)"])

)

write.table(outTab, file= "test_7_cox.txt", sep="\t", row.names=F, quote=F)

####

#### test_8

####

rm(list = ls())

data=read.table("test_8_risk.txt",header=T,sep="\t",check.names=F)

rt <- data[,c(2,3,11)]

colnames(rt)[3] <- c("riskScore")

rownames(rt) <- data$id

source("survivalROC_NEW.R")

cutoffROC <- function(t,rt){

rt=rt

ROC_rt <- survivalROC_NEW(Stime=rt[,1], status=rt[,2], marker = rt[,3],

predict.time =t, method="KM")

youdenindex=max(ROC_rt$sensitivity+ROC_rt$specificity-1)

index = which(ROC_rt$sensitivity+ROC_rt$specificity-1==youdenindex)+1

result=c(ROC_rt$cut.values[index],ROC_rt$sensitivity[index-1],ROC_rt$specificity[index-1])

}

####

t <- 4

####

(res.cut <- cutoffROC(t,rt))

cutoff <- res.cut[1]

save(cutoff,file = "cutoff_test_8.Rdata")

####

gene <- "riskScore"

rt <- rt[,c("futime","fustat",gene)]

rt$risk <- ifelse(rt$riskScore > cutoff,"high","low")

table(rt$risk)

####

my.surv <- Surv(rt$futime, rt$fustat)

group <- rt$risk

survival_dat <- data.frame(group = group)

fit <- survfit(my.surv ~ group)

summary(fit)$table

sur_high <- round(summary(fit)$table[1,7],3)

sur_low <- round(summary(fit)$table[2,7],3)

median <- c(sur_high,sur_low)

if(T){

group <- factor(group, levels = c("low", "high"))

data.survdiff <- survdiff(my.surv ~ group)

p.val = 1 - pchisq(data.survdiff$chisq, length(data.survdiff$n) - 1)

x = summary(coxph(Surv(futime, fustat)~riskScore, data = rt))

HR = signif(x$coef[2], digits=3)

up95 = signif(x$conf.int[,"upper .95"],3)

low95 = signif(x$conf.int[,"lower .95"], 3)

HR <- paste("HR = ", round(HR,3), sep = "")

CI <- paste(paste(round(low95,3), round(up95,3), sep = " - "), sep = "")

rt <- rt[order(rt[,"riskScore"],decreasing = T),]

ggsurvplot(fit, data = survival_dat ,

ggtheme = theme_bw(),

conf.int = T,

conf.int.style = "ribbon",

censor = T,

break.time.by = 2,

surv.median.line = "hv",

palette = c("darkorange","deepskyblue"),

ncensor.plot = FALSE,

font.legend = 12,

pval = paste(paste("pvalue =", p.val), paste(HR,"(",CI,")"),

paste("Median OS = ",median[1]," vs ",median[2]),sep = "\n"))

}

library(export)

graph2ppt(file= "test_8_sur.ppt",width=7,height=6)

####

#### HR

####

library("survival")

library("survminer")

gene <- colnames(rt)[3]

library(tibble)

library(dplyr)

rt1 <- rt %>%

select(futime,fustat,gene)

outTab=data.frame()

cox=coxph(Surv(futime, fustat) ~ rt1[,3], data = rt1)

coxSummary=summary(cox)

outTab=rbind(outTab,

cbind(id="test 8",

HR=coxSummary$conf.int[,"exp(coef)"],

HR.95L=coxSummary$conf.int[,"lower .95"],

HR.95H=coxSummary$conf.int[,"upper .95"],

pvalue=coxSummary$coefficients[,"Pr(>|z|)"])

)

write.table(outTab, file= "test_8_cox.txt", sep="\t", row.names=F, quote=F)

####

#### meta

####

rm(list = ls())

library(dplyr)

library(ggpubr)

library(meta)

files=dir()

files=grep("_cox.txt",files,value=T)

data=data.frame()

for(i in files){

rt=read.table(i, header=T, sep="\t", check.names=F)

data=rbind(data, rt)

}

study=data$id

HR=data$HR

lower.HR=data$HR.95L

upper.HR=data$HR.95H

meta=metagen(log(HR),

lower=log(lower.HR),

upper=log(upper.HR),

studlab = study,

sm = "HR",

comb.random=TRUE,

comb.fixed=FALSE)

####

meta

####

forest(meta,

col.square = "green",

col.diamond = "red",

col.diamond.lines = "red",

xlim = c(0.8, 4))

library(export)

graph2ppt(file = paste0("meta.ppt"),width=10,height=14)

####

#### clinical feature

####

rm(list = ls())

exp <- read.table("train_risk.txt",header=T,sep="\t",check.names=F)

cli <- read.table("clinical_2.txt",header=T,sep="\t",check.names=F)

same_sample <- intersect(cli$id,exp$id)

library(dplyr)

library(tibble)

cli <- cli[cli$id %in% same_sample,]

exp <- exp[exp$id %in% same_sample,]

cli_exp <- exp %>%

inner_join(cli, by = "id") %>%

column_to_rownames("id")

rt <- cli_exp[,c(10,12:17)]

Age <- rt[,c(1:2)]

Age <- na.omit(Age)

age_less_65 <- Age[Age$age == "<65",]

age_more_65 <- Age[Age$age == ">65",]

Age <- rbind(age_less_65,age_more_65)

colnames(Age) <- c("riskScore","clinical")

####

Gender <- rt[,c(1,3)]

Gender <- na.omit(Gender)

female <- Gender[Gender$gender == "female",]

male <- Gender[Gender$gender == "male",]

Gender <- rbind(female,male)

colnames(Gender) <- c("riskScore","clinical")

####

Stage <- rt[,c(1,4)]

Stage <- na.omit(Stage)

I_II <- Stage[Stage$stage == "I_II",]

III_IV <- Stage[Stage$stage == "III_IV",]

Stage <- rbind(I_II,III_IV)

colnames(Stage) <- c("riskScore","clinical")

####

T_T <- rt[,c(1,5)]

T_T <- na.omit(T_T)

T1 <- T_T[T_T$pathologic_T == "T1_T2",]

T2 <- T_T[T_T$pathologic_T == "T3_T4",]

T_T <- rbind(T1,T2)

colnames(T_T) <- c("riskScore","clinical")

####

N <- rt[,c(1,6)]

N <- na.omit(N)

N0 <- N[N$pathologic_N == "N0",]

N1 <- N[N$pathologic_N == "N1_N2_N3",]

N <- rbind(N0,N1)

colnames(N) <- c("riskScore","clinical")

####

M <- rt[,c(1,7)]

M <- na.omit(M)

M0 <- M[M$pathologic_M == "M0",]

M1 <- M[M$pathologic_M == "M1",]

M <- rbind(M0,M1)

colnames(M) <- c("riskScore","clinical")

####

data <- rbind(Age,Gender,Stage,T_T,N,M)

data$clinical <- factor(data$clinical,levels = c("<65",">65","female","male","I_II","III_IV","T1_T2",

"T3_T4","N0","N1_N2_N3","M0","M1"))

table(data$clinical)

boxplot(riskScore ~ clinical, data = data,

ylab = paste("expression",sep=""),

col = c("#00AFBB", "#00AFBB","slateblue1", "slateblue1",

"blue","blue","orange","orange",

"green","green","tomato","tomato","magenta","magenta"),

cex.main=1.5, cex.lab=1.3, cex.axis=1.2,ylim=c(0,3.45),outline = FALSE)

library(export)

graph2ppt(file = "clinical_1.txt",width=19,height=7)

####

#### Univariate analysis and multivariate analysis

####

rm(list = ls())

library(survival)

exp <- read.table("train_risk.txt",header=T,sep="\t",check.names=F,row.names = 1)

clinical <- read.table("clinical.txt",header=T,sep="\t",check.names=F,row.names = 1)

same <- intersect(rownames(exp),rownames(clinical))

exp <- exp[same,]

clinical <- clinical[same,]

library(tibble)

library(dplyr)

exp <- exp %>%

rownames_to_column("id")

rt <- clinical %>%

rownames_to_column("id") %>%

inner_join(exp, by = "id")

rt <- rt %>%

select("id","futime","fustat","Age","Gender","Stage",

"T_classification","N_classification","M_classification","riskScore") %>%

column_to_rownames("id")

uniTab=data.frame()

for(i in colnames(rt[,3:ncol(rt)])){

cox <- coxph(Surv(futime, fustat) ~ rt[,i], data = rt)

coxSummary = summary(cox)

uniTab=rbind(uniTab,

cbind(id=i,

HR=coxSummary$conf.int[,"exp(coef)"],

HR.95L=coxSummary$conf.int[,"lower .95"],

HR.95H=coxSummary$conf.int[,"upper .95"],

pvalue=coxSummary$coefficients[,"Pr(>|z|)"])

)

}

write.table(uniTab,file = "uniCox.txt",sep="\t",row.names=F,quote=F)

uniTab=uniTab[as.numeric(as.character(uniTab[,"pvalue"])) <0.05,]

rt1=rt[,c("futime","fustat",as.vector(uniTab[,"id"]))]

multiCox=coxph(Surv(futime, fustat) ~ ., data = rt1)

multiCoxSum=summary(multiCox)

multiTab=data.frame()

multiTab=cbind(

HR=multiCoxSum$conf.int[,"exp(coef)"],

HR.95L=multiCoxSum$conf.int[,"lower .95"],

HR.95H=multiCoxSum$conf.int[,"upper .95"],

pvalue=multiCoxSum$coefficients[,"Pr(>|z|)"])

multiTab=cbind(id=row.names(multiTab),multiTab)

write.table(multiTab,file = "multiCox.txt",sep="\t",row.names=F,quote=F)

bioForest=function(coxFile=null,forestFile=null,height=null,forestCol=null){

rt <- read.table(coxFile,header=T,sep="\t",row.names=1,check.names=F)

gene <- rownames(rt)

hr <- sprintf("%.3f",rt$"HR")

hrLow <- sprintf("%.3f",rt$"HR.95L")

hrHigh <- sprintf("%.3f",rt$"HR.95H")

Hazard.ratio <- paste0(hr,"(",hrLow,"-",hrHigh,")")

pVal <- ifelse(rt$pvalue<0.001, "<0.001", sprintf("%.3f", rt$pvalue))

n <- nrow(rt)

nRow <- n+1

ylim <- c(1,nRow)

layout(matrix(c(1,2),nc=2),width=c(3,2.5))

####

xlim = c(0,3)

par(mar=c(4,2.5,2,1))

plot(1,xlim=xlim,ylim=ylim,type="n",axes=F,xlab="",ylab="")

text.cex=0.8

text(0,n:1,gene,adj=0,cex=text.cex)

text(1.5-0.5*0.2,n:1,pVal,adj=1,cex=text.cex);text(1.5-0.5*0.2,n+1,'pvalue',cex=text.cex,adj=1)

text(3,n:1,Hazard.ratio,adj=1,cex=text.cex);text(3,n+1,'Hazard ratio',cex=text.cex,adj=1,)

####

par(mar=c(4,1,2,1),mgp=c(2,0.5,0))

xlim = c(0,max(as.numeric(hrLow),as.numeric(hrHigh)))

plot(1,xlim=xlim,ylim=ylim,type="n",axes=F,ylab="",xaxs="i",xlab="Hazard ratio")

arrows(as.numeric(hrLow),n:1,as.numeric(hrHigh),n:1,angle=90,code=3,length=0.05,col="darkblue",lwd=2.5)

abline(v=1,col="black",lty=2,lwd=2)

boxcolor = ifelse(as.numeric(hr) > 1, forestCol[1], forestCol[2])

points(as.numeric(hr), n:1, pch = 15, col = boxcolor, cex=1.6)

axis(1)

}

####

bioForest(coxFile="uniCox.txt",forestFile="uniForest.pdf", forestCol=c("green","green"))

library(export)

graph2ppt(file = "uniCox.pptx",width=8.5, height=4.5)

####

bioForest(coxFile="multiCox.txt",forestFile="multiForest.pdf",forestCol=c("red","red"))

library(export)

graph2ppt(file = "multiCox.pptx",width=8.5, height=4.5)

####

#### nomogram

####

rm(list = ls())

library(survival)

exp <- read.table("train_risk.txt",header=T,sep="\t",check.names=F,row.names = 1)

clinical <- read.table("clinical.txt",header=T,sep="\t",check.names=F,row.names = 1)

####

same <- intersect(rownames(exp),rownames(clinical))

####

exp <- exp[same,]

clinical <- clinical[same,]

library(tibble)

library(dplyr)

exp <- exp %>%

rownames_to_column("id")

rt <- clinical %>%

rownames_to_column("id") %>%

inner_join(exp, by = "id")

rt <- rt %>%

select("id","futime","fustat","Age","Gender","Stage",

"T_classification","N_classification","M_classification","riskScore") %>%

column_to_rownames("id")

write.table(rt,file = "rt.xls",sep = "\t",row.names = T,col.names = T)

####

rt <- read.table("rt.txt",check.names = F,row.names = 1,header=T,sep="\t",)

library(regplot)

library(survival)

library(survminer)

head(rt)

rt$futime <- as.numeric(rt$futime)

rt$fustat <- as.numeric(rt$fustat)

rt$Gender <- as.numeric(rt$Gender)

rt$Stage <- as.numeric(rt$Stage)

rt$T_classification <- as.numeric(rt$T_classification)

rt$N_classification <- as.numeric(rt$N_classification)

rt$M_classification <- as.numeric(rt$M_classification)

rt$riskScore <- as.numeric(rt$riskScore)

rt$Age <- as.factor(ifelse(rt$Age == 1, "≤65",">65"))

rt$Gender <- as.factor(ifelse(rt$Gender == 1, "Female","Male"))

rt$M_classification <- as.factor(ifelse(rt$M_classification == 1, "M0","M1"))

res.cox <- coxph(Surv(futime, fustat) ~ Age + Gender + Stage + T_classification + N_classification + M_classification +riskScore, data = rt)

nom1 <- regplot(res.cox,clickable = TRUE,

points = TRUE, rank = "sd", failtime = c(1,2,3,4),prfail = T)

library(export)

graph2ppt(file = "Nomogram.ppt",width=12, height=10)

####

#### Calibration curves

####

rm(list = ls())

rt <- read.table("rt.txt",check.names = F,row.names = 1,header=T,sep="\t",)

library(rms)

dd <- datadist(rt)

options(datadist="dd")

####

time=1

f_1 <- cph(Surv(futime, fustat) ~ Age + Gender + Stage + T_classification + N_classification + M_classification +riskScore,

x=T, y=T, surv=T, data=rt, time.inc=time)

P_1 <- calibrate(f_1, cmethod="KM", method="boot", u=time, m=111, B=1000)

plot(P_1,

add = F,

subtitles = F,

cex.subtitles = 0.8,

lwd = 2,

lty = 1,

errbar.col = "red",

xlim = c(0,1),

ylim = c(0,1),

xlab="Nomogram-Predicted Probability of 1, 2, 3, 4-Year OS",

ylab="Actual 1, 2, 3, 4-Year OS(proportion)",

col="red",

sub=F)

####

time=2

f_2 <- cph(Surv(futime, fustat) ~ Age + Gender + Stage + T_classification + N_classification + M_classification +riskScore,

x=T, y=T, surv=T, data=rt, time.inc=time)

P_2 <- calibrate(f_2, cmethod="KM", method="boot", u=time, m=111, B=1000)

plot(P_2,

add = T,

subtitles = F,

cex.subtitles = 0.8,

lwd = 2,

lty = 1,

errbar.col = "orange",

xlim = c(0,1),

ylim = c(0,1),

xlab="Nomogram-Predicted Probability of 1, 2, 3, 4-Year OS",

ylab="Actual 1, 2, 3, 4-Year OS(proportion)",

col="orange",

sub=F)

####

time=3

f_3 <- cph(Surv(futime, fustat) ~ Age + Gender + Stage + T_classification + N_classification + M_classification +riskScore,

x=T, y=T, surv=T, data=rt, time.inc=time)

P_3 <- calibrate(f_3, cmethod="KM", method="boot", u=time, m=111, B=1000)

plot(P_3,

add = T,

subtitles = F,

cex.subtitles = 0.8,

lwd = 2,

lty = 1,

errbar.col = "forestgreen",

xlim = c(0,1),

ylim = c(0,1),

xlab="Nomogram-Predicted Probability of 1, 2, 3, 4-Year OS",

ylab="Actual 1, 2, 3, 4-Year OS(proportion)",

col="forestgreen",

sub=F)

####

time=4

f_4 <- cph(Surv(futime, fustat) ~ Age + Gender + Stage + T_classification + N_classification + M_classification +riskScore,

x=T, y=T, surv=T, data=rt, time.inc=time)

P_4 <- calibrate(f_4, cmethod="KM", method="boot", u=time, m=111, B=1000)

plot(P_4,

add = T,

subtitles = F,

cex.subtitles = 0.8,

lwd = 2,

lty = 1,

errbar.col = "blue",

xlim = c(0,1),

ylim = c(0,1),

xlab="Nomogram-Predicted Probability of 1, 2, 3, 4-Year OS",

ylab="Actual 1, 2, 3, 4-Year OS(proportion)",

col="blue",

sub=F)

####

legend("bottomright",legend = c("1 year","2 year","3 year","4 year"),

col = c("red","orange","forestgreen","blue"),lwd = 2)

library(export)

graph2ppt(file = "alignment chart.ppt",width=5, height=5)

####

rm(list = ls())

compare <- read.table("compare.txt",header=T,sep="\t",check.names=F)

library(ggplot2)

data <- compare

library(tidyr)

data <- data %>%

pivot_longer(cols=-1,

names_to= "gene",

values_to = "expression")

p <- ggplot(data=data, aes(x=gene,y=expression,fill=model)) +

geom_bar(stat="identity", color="black", position=position_dodge())+

theme_minimal()

p + scale_fill_manual(values=c('white','#E69F00','green','gray'))

library(export)

graph2ppt(file = "compare.ppt",width=6,height=4.5)

####

#### ROC curves

####

rm(list = ls())

rt <- read.table("rt.txt",check.names = F,row.names = 1,header=T,sep="\t",)

library(survival)

library(survminer)

library(timeROC)

library(survivalROC)

####

aucText=c()

rocCol=rainbow(ncol(rt)-1)

gene=colnames(rt)[9]

roc=survivalROC(Stime=rt$futime, status=rt$fustat, marker = rt$riskScore, predict.time =1, method="KM")

plot(roc$FP, roc$TP, type="l", xlim=c(0,1), ylim=c(0,1),col=rocCol[1],

xlab="False positive rate", ylab="True positive rate",

lwd = 2, cex.main=1.3, cex.lab=1.2, cex.axis=1.2, font=1.2)

aucText=c(aucText,paste0("riskScore"," (AUC=",sprintf("%.3f",roc$AUC),")"))

abline(0,1)

####

j=1

for(i in colnames(rt[,3:(ncol(rt)-1)])){

roc=survivalROC(Stime=rt$futime, status=rt$fustat, marker = rt[,i], predict.time =1, method="KM")

j=j+1

aucText=c(aucText,paste0(i," (AUC=",sprintf("%.3f",roc$AUC),")"))

lines(roc$FP, roc$TP, type="l", xlim=c(0,1), ylim=c(0,1),col=rocCol[j],lwd = 2)

}

legend("bottomright", aucText,lwd=2,bty="n",col=rocCol)

library(export)

graph2ppt(file = "ROC_1.ppt",width=5, height=5)

####

#### 2 years

####

rm(list = ls())

rt <- read.table("rt.txt",check.names = F,row.names = 1,header=T,sep="\t",)

library(survival)

library(survminer)

library(timeROC)

library(survivalROC)

####

aucText=c()

rocCol=rainbow(ncol(rt)-1)

gene=colnames(rt)[9]

roc=survivalROC(Stime=rt$futime, status=rt$fustat, marker = rt$riskScore, predict.time =2, method="KM")

plot(roc$FP, roc$TP, type="l", xlim=c(0,1), ylim=c(0,1),col=rocCol[1],

xlab="False positive rate", ylab="True positive rate",

lwd = 2, cex.main=1.3, cex.lab=1.2, cex.axis=1.2, font=1.2)

aucText=c(aucText,paste0("riskScore"," (AUC=",sprintf("%.3f",roc$AUC),")"))

abline(0,1)

####

j=1

for(i in colnames(rt[,3:(ncol(rt)-1)])){

roc=survivalROC(Stime=rt$futime, status=rt$fustat, marker = rt[,i], predict.time =2, method="KM")

j=j+1

aucText=c(aucText,paste0(i," (AUC=",sprintf("%.3f",roc$AUC),")"))

lines(roc$FP, roc$TP, type="l", xlim=c(0,1), ylim=c(0,1),col=rocCol[j],lwd = 2)

}

legend("bottomright", aucText,lwd=2,bty="n",col=rocCol)

library(export)

graph2ppt(file = "ROC_2.ppt",width=5, height=5)

####

#### 3 years

####

rm(list = ls())

rt <- read.table("rt.txt",check.names = F,row.names = 1,header=T,sep="\t",)

library(survival)

library(survminer)

library(timeROC)

library(survivalROC)

####

aucText=c()

rocCol=rainbow(ncol(rt)-1)

gene=colnames(rt)[9]

roc=survivalROC(Stime=rt$futime, status=rt$fustat, marker = rt$riskScore, predict.time =3, method="KM")

plot(roc$FP, roc$TP, type="l", xlim=c(0,1), ylim=c(0,1),col=rocCol[1],

xlab="False positive rate", ylab="True positive rate",

lwd = 2, cex.main=1.3, cex.lab=1.2, cex.axis=1.2, font=1.2)

aucText=c(aucText,paste0("riskScore"," (AUC=",sprintf("%.3f",roc$AUC),")"))

abline(0,1)

####

j=1

for(i in colnames(rt[,3:(ncol(rt)-1)])){

roc=survivalROC(Stime=rt$futime, status=rt$fustat, marker = rt[,i], predict.time =3, method="KM")

j=j+1

aucText=c(aucText,paste0(i," (AUC=",sprintf("%.3f",roc$AUC),")"))

lines(roc$FP, roc$TP, type="l", xlim=c(0,1), ylim=c(0,1),col=rocCol[j],lwd = 2)

}

legend("bottomright", aucText,lwd=2,bty="n",col=rocCol)

library(export)

graph2ppt(file = "ROC_3.ppt",width=5, height=5)

####

#### 4 years

####

rm(list = ls())

rt <- read.table("rt.txt",check.names = F,row.names = 1,header=T,sep="\t",)

library(survival)

library(survminer)

library(timeROC)

library(survivalROC)

####

aucText=c()

rocCol=rainbow(ncol(rt)-1)

gene=colnames(rt)[9]

roc=survivalROC(Stime=rt$futime, status=rt$fustat, marker = rt$riskScore, predict.time =4, method="KM")

plot(roc$FP, roc$TP, type="l", xlim=c(0,1), ylim=c(0,1),col=rocCol[1],

xlab="False positive rate", ylab="True positive rate",

lwd = 2, cex.main=1.3, cex.lab=1.2, cex.axis=1.2, font=1.2)

aucText=c(aucText,paste0("riskScore"," (AUC=",sprintf("%.3f",roc$AUC),")"))

abline(0,1)

####

j=1

for(i in colnames(rt[,3:(ncol(rt)-1)])){

roc=survivalROC(Stime=rt$futime, status=rt$fustat, marker = rt[,i], predict.time =4, method="KM")

j=j+1

aucText=c(aucText,paste0(i," (AUC=",sprintf("%.3f",roc$AUC),")"))

lines(roc$FP, roc$TP, type="l", xlim=c(0,1), ylim=c(0,1),col=rocCol[j],lwd = 2)

}

legend("bottomright", aucText,lwd=2,bty="n",col=rocCol)

library(export)

graph2ppt(file = "ROC_4.ppt",width=5, height=5)

####

#### GSEA

####

rm(list = ls())

load("TCGA_LUAD_59Normal_513Tumor.Rdata")

risk <- read.table("train_risk.txt",header = T,sep="\t",check.names=F,row.names = 1)

risk <- risk[,c(10,11)]

exprSet4 <- exprSet1[exprSet1$sample == "Tumor",]

exprSet4 <- exprSet4[,-1]

same_sample <- intersect(rownames(exprSet4),rownames(risk))

risk <- risk[same_sample,]

exprSet <- exprSet4[same_sample,]

library(tibble)

library(dplyr)

exprSet1 <- exprSet %>%

rownames_to_column("id") %>%

inner_join((risk <- risk %>%

rownames_to_column("id")),by = "id") %>%

column_to_rownames("id") %>%

dplyr::select(riskScore,risk,everything())

test <- exprSet1[,1:20]

low_risk <- exprSet1[exprSet1$risk == "low",]

high_risk <- exprSet1[exprSet1$risk == "high",]

data <- as.data.frame(rbind(low_risk,high_risk))

test <- data[,1:20]

save(data, file = "exprSet1.Rdata")

####

library(limma)

exprSet <- as.data.frame(t(data[,-c(1,2)]))

group <- c(rep("low",259),rep("high",241))

group <- factor(group,levels = c("low","high"),ordered = F)

design <- model.matrix(~group)

colnames(design) <- levels(group)

fit <- lmFit(exprSet,design)

fit2 <- eBayes(fit)

####

all_Diff_gene = topTable(fit2,adjust='fdr',coef=2,number=Inf)

rt <- all_Diff_gene

library(tibble)

library(dplyr)

rt <- rt %>%

rownames_to_column("gene")

gene <- rt$gene

library(clusterProfiler)

gene = bitr(gene, fromType="SYMBOL", toType="ENTREZID", OrgDb="org.Hs.eg.db")

gene <- dplyr::distinct(gene,SYMBOL,.keep_all=TRUE)

gene_df <- data.frame(logFC=rt$logFC,SYMBOL = rt$gene)

gene_df <- merge(gene_df,gene,by="SYMBOL")

colnames(gene_df) <- c("gene","logFC","entrez")

geneList <- gene_df$logFC

names(geneList) = gene_df$gene

geneList = sort(geneList, decreasing = TRUE)

head(geneList)

####

library(clusterProfiler)

hallmarks_kegg <- read.gmt("c2.cp.kegg.v7.4.symbols.gmt")

kegg <- GSEA(geneList,TERM2GENE = hallmarks_kegg,pvalueCutoff = 1)

kegg_show <- data.frame(kegg)

yd_kegg <- data.frame(kegg_show)

####

write.table(yd_kegg, file = "KEGG.xls", sep="\t",row.names = F,quote=F, col.names = T)

####

enrich_num <- stringr::str_count(yd_kegg$core_enrichment,"/")+1

yd_kegg$GeneRatio <- as.numeric(enrich_num/yd_kegg$setSize)

yd_kegg$ID <- substring(yd_kegg$ID,10)

data <- yd_kegg

####

library(ggplot2)

library(ggrepel)

ggplot(data=data, aes(x=NES, y = -log10(p.adjust))) +

geom_point(data=subset(data,data$p.adjust>=0.05),aes(size=GeneRatio),alpha=0.3)+

geom_point(data=subset(data,data$p.adjust<0.05 & data$NES > 1),aes(size=abs(GeneRatio)),color= "orange",alpha=0.9) +

geom_point(data=subset(data,data$p.adjust<0.05 & data$NES < -1),aes(size=abs(GeneRatio)),color= "green",alpha=0.9) +

geom_hline(yintercept = -log10(0.05),lty=4,lwd=1.2,alpha=0.8)+

theme_bw()+

theme(panel.border = element_blank(),

panel.grid.major = element_blank(),

panel.grid.minor = element_blank(),

axis.line = element_line(colour = "black")) +

geom_text_repel(data=subset(data, rownames(data) == "KEGG_CELL_CYCLE"),

aes(label= Description),col="black",alpha = 0.8,direction = "y",angle = 90,vjust = 1.5)+

geom_text_repel(data=subset(data, rownames(data) == "KEGG_DNA_REPLICATION"),

aes(label= Description),col="black",alpha = 0.8,direction = "x",angle = 0,vjust = 1.5)+

geom_text_repel(data=subset(data, rownames(data) == "KEGG_P53_SIGNALING_PATHWAY"),

aes(label= Description),col="black",alpha = 0.8,direction = "x",angle = 90,vjust = 1.5)+

geom_text_repel(data=subset(data, rownames(data) == "KEGG_DRUG_METABOLISM_CYTOCHROME_P450"),

aes(label= Description),col="black",alpha = 0.8,direction = "x",angle = 90,vjust = 1.5)+

geom_text_repel(data=subset(data, rownames(data) == "KEGG_CELL_ADHESION_MOLECULES_CAMS"),

aes(label= Description),col="black",alpha = 0.8,direction = "x",angle = 90,vjust = 1.5)+

geom_text_repel(data=subset(data, rownames(data) == "KEGG_METABOLISM_OF_XENOBIOTICS_BY_CYTOCHROME_P450"),

aes(label= Description),col="black",alpha = 0.8,direction = "y",angle = 90,vjust = 1.5)+

geom_text_repel(data=subset(data, rownames(data) == "KEGG_ANTIGEN_PROCESSING_AND_PRESENTATION"),

aes(label= Description),col="black",alpha = 0.8,direction = "x",angle = 45,vjust = 1.5)+

geom_text_repel(data=subset(data, rownames(data) == "KEGG_NATURAL_KILLER_CELL_MEDIATED_CYTOTOXICITY"),

aes(label= Description),col="black",alpha = 0.8,direction = "x",angle = 90,vjust = 1.5)+

geom_text_repel(data=subset(data, rownames(data) == "KEGG_T_CELL_RECEPTOR_SIGNALING_PATHWAY"),

aes(label= Description),col="black",alpha = 0.8,direction = "x",angle = 45,vjust = 1.5)+

geom_text_repel(data=subset(data, rownames(data) == "KEGG_B_CELL_RECEPTOR_SIGNALING_PATHWAY"),

aes(label= Description),col="black",alpha = 0.8,direction = "x",angle = 90,vjust = 1.5)+

geom_text_repel(data=subset(data, rownames(data) == "KEGG_CHEMOKINE_SIGNALING_PATHWAY"),

aes(label= Description),col="black",alpha = 0.8,direction = "y",angle = 90,vjust = 1.5)+

geom_text_repel(data=subset(data, rownames(data) == "KEGG_CYTOKINE_CYTOKINE_RECEPTOR_INTERACTION"),

aes(label= Description),col="black",alpha = 0.8,direction = "x",angle = 45,vjust = 1.5)+

geom_text_repel(data=subset(data, rownames(data) == "KEGG_JAK_STAT_SIGNALING_PATHWAY"),

aes(label= Description),col="black",alpha = 0.8,direction = "x",angle = 90,vjust = 1.5)+

geom_text_repel(data=subset(data, rownames(data) == "KEGG_LEUKOCYTE_TRANSENDOTHELIAL_MIGRATION"),

aes(label= Description),col="black",alpha = 0.8,direction = "x",angle = 45,vjust = 1.5)+

geom_text_repel(data=subset(data, rownames(data) == "KEGG_PATHWAYS_IN_CANCER"),

aes(label= Description),col="black",alpha = 0.8,direction = "x",angle = 90,vjust = 1.5)

####

library(export)

graph2ppt(file = paste0("KEGG.ppt"), width=9, height=7)

####

####

####

rt <- all_Diff_gene

library(tibble)

library(dplyr)

rt <- rt %>%

rownames_to_column("gene")

gene <- rt$gene

library(clusterProfiler)

gene = bitr(gene, fromType="SYMBOL", toType="ENTREZID", OrgDb="org.Hs.eg.db")

gene <- dplyr::distinct(gene,SYMBOL,.keep_all=TRUE)

gene_df <- data.frame(logFC=rt$logFC,SYMBOL = rt$gene)

gene_df <- merge(gene_df,gene,by="SYMBOL")

colnames(gene_df) <- c("gene","logFC","entrez")

geneList <- gene_df$logFC

names(geneList) = gene_df$gene

geneList = sort(geneList, decreasing = TRUE)

head(geneList)

####

library(clusterProfiler)

hallmarks_GO_BP <- read.gmt("c5.go.bp.v7.4.symbols.gmt")

GO_BP <- GSEA(geneList,TERM2GENE = hallmarks_GO_BP, pvalueCutoff = 1)

GO_BP_show <- data.frame(GO_BP)

yd_GO_BP <- data.frame(GO_BP_show)

####

write.table(yd_GO_BP, file = "GO_BP.xls", sep="\t",row.names = F,quote=F, col.names = T)

enrich_num <- stringr::str_count(yd_GO_BP$core_enrichment,"/")+1

yd_GO_BP$GeneRatio <- as.numeric(enrich_num/yd_GO_BP$setSize)

yd_GO_BP$ID <- substring(yd_GO_BP$ID,10)

data <- yd_GO_BP

library(ggplot2)

library(ggrepel)

ggplot(data=data, aes(x= NES, y = -log10(p.adjust))) +

geom_point(data=subset(data,data$p.adjust>=0.05),aes(size=GeneRatio),alpha=0.3)+

geom_point(data=subset(data,data$p.adjust<0.05 & data$NES > 1),aes(size=abs(GeneRatio)),color= "orange",alpha=0.9) +

geom_point(data=subset(data,data$p.adjust<0.05 & data$NES < -1),aes(size=abs(GeneRatio)),color= "green",alpha=0.9) +

geom_hline(yintercept = -log10(0.05),lty=4,lwd=0.6,alpha=0.8)+

theme_bw()+

theme(panel.border = element_blank(),

panel.grid.major = element_blank(),

panel.grid.minor = element_blank(),

axis.line = element_line(colour = "black")) +

geom_text_repel(data=subset(data, rownames(data) == "GOBP_ADAPTIVE_IMMUNE_RESPONSE"),

aes(label= Description),col="black",alpha = 0.8,direction = "y",angle = 90,vjust = 1.5)+

geom_text_repel(data=subset(data, rownames(data) == "GOBP_ANTIGEN_PROCESSING_AND_PRESENTATION"),

aes(label= Description),col="black",alpha = 0.8,direction = "x",angle = 0,vjust = 1.5)+

geom_text_repel(data=subset(data, rownames(data) == "GOBP_ACTIVATION_OF_IMMUNE_RESPONSE"),

aes(label= Description),col="black",alpha = 0.8,direction = "x",angle = 90,vjust = 1.5)+

geom_text_repel(data=subset(data, rownames(data) == "GOBP_B_CELL_ACTIVATION"),

aes(label= Description),col="black",alpha = 0.8,direction = "x",angle = 90,vjust = 1.5)+

geom_text_repel(data=subset(data, rownames(data) == "GOBP_CD4_POSITIVE_ALPHA_BETA_T_CELL_ACTIVATION"),

aes(label= Description),col="black",alpha = 0.8,direction = "x",angle = 90,vjust = 1.5)+

geom_text_repel(data=subset(data, rownames(data) == "GOBP_CD8_POSITIVE_ALPHA_BETA_T_CELL_ACTIVATION"),

aes(label= Description),col="black",alpha = 0.8,direction = "y",angle = 30,vjust = 1.5)+

geom_text_repel(data=subset(data, rownames(data) == "GOBP_DENDRITIC_CELL_ANTIGEN_PROCESSING_AND_PRESENTATION"),

aes(label= Description),col="black",alpha = 0.8,direction = "x",angle = 90,vjust = 1.5)

geom_text_repel(data=subset(data, rownames(data) == "GOBP_LEUKOCYTE_MEDIATED_CYTOTOXICITY"),

aes(label= Description),col="black",alpha = 0.8,direction = "x",angle = 45,vjust = 1.5)+

geom_text_repel(data=subset(data, rownames(data) == "GOBP_LYMPHOCYTE_MEDIATED_IMMUNITY"),

aes(label= Description),col="black",alpha = 0.8,direction = "x",angle = 45,vjust = 1.5)+

geom_text_repel(data=subset(data, rownames(data) == "GOBP_MACROPHAGE_ACTIVATION"),

aes(label= Description),col="black",alpha = 0.8,direction = "x",angle = 90,vjust = 1.5)+

geom_text_repel(data=subset(data, rownames(data) == "GOBP_HUMORAL_IMMUNE_RESPONSE"),

aes(label= Description),col="black",alpha = 0.8,direction = "x",angle = 90,vjust = 1.5)+

geom_text_repel(data=subset(data, rownames(data) == "GOBP_MAST_CELL_ACTIVATION_INVOLVED_IN_IMMUNE_RESPONSE"),

aes(label= Description),col="black",alpha = 0.8,direction = "x",angle = 45,vjust = 1.5)+

geom_text_repel(data=subset(data, rownames(data) == "GOBP_NATURAL_KILLER_CELL_ACTIVATION"),

aes(label= Description),col="black",alpha = 0.8,direction = "x",angle = 45,vjust = 1.5)+

geom_text_repel(data=subset(data, rownames(data) == "GOBP_T_CELL_ACTIVATION"),

aes(label= Description),col="black",alpha = 0.8,direction = "x",angle = 90,vjust = 1.5)+

geom_text_repel(data=subset(data, rownames(data) == "GOBP_T_CELL_RECEPTOR_SIGNALING_PATHWAY"),

aes(label= Description),col="black",alpha = 0.8,direction = "x",angle = 0,vjust = 1.5)+

geom_text_repel(data=subset(data, rownames(data) == "GOBP_B_CELL_RECEPTOR_SIGNALING_PATHWAY"),

aes(label= Description),col="black",alpha = 0.8,direction = "x",angle = 0,vjust = 1.5)

####

library(export)

graph2ppt(file = paste0("GO_BP.ppt"), width=9.5, height=7)

####

#### cibersort

####

rm(list = ls())

library(limma)

library(estimate)

load(file = "exprSet1.Rdata")

test <- data[1:20,1:20]

exprSet3 <- as.data.frame(t(data[,-c(1,2)]))

library(tibble)

library(dplyr)

exprSet3 <- exprSet3 %>%

rownames_to_column("ID")

test <- exprSet3[1:20,1:20]

write.table(exprSet3, file="uniq.symbol.txt", sep="\t", quote=F, col.names=T, row.names = F)

####

filterCommonGenes(input.f="uniq.symbol.txt",

output.f="commonGenes.gct",

id="GeneSymbol")

####

estimateScore(input.ds="commonGenes.gct",

output.ds="estimateScore.gct")

####

scores <- read.table("estimateScore.gct", skip=2, header=T, check.names=F)

rownames(scores) <- scores[,1]

scores <- t(scores[,3:ncol(scores)])

rownames(scores) <- gsub("\\.", "\\-", rownames(scores))

scores <- as.data.frame(scores[,1:3])

save(scores,file = "scores.Rdata")

####

rm(list = ls())

load("exprSet1.Rdata")

exp <- data[,c(1,2)]

load("scores.Rdata")

same <- intersect(rownames(scores),rownames(exp))

library(tibble)

library(dplyr)

exp1 <- exp %>%

rownames_to_column("id") %>%

inner_join(scores <- scores %>%

rownames_to_column("id"), by = "id") %>%

column_to_rownames("id")

save(exp1, file = "exp1.Rdata")

####

rm(list = ls())

load("exp1.Rdata")

library(ggplot2)

library(ggpubr)

data <- exp1

data <- data[,-1]

library(tidyr)

data <- data %>%

pivot_longer(cols=-1,

names_to= "gene",

values_to = "expression")

ggplot(data = data,aes(x=gene,y=expression,fill=risk))+

geom_boxplot()+

theme_bw()+

stat_compare_means(label = "p.format")

library(export)

graph2ppt(file = "scpre.txt",width=5,height=4.5)

####

#### ssGSEA

####

rm(list=ls())

load(file = "cellMarker_ssGSEA.Rdata")

load(file = "exprSet1.Rdata")

exprSet3 <- data

expr <- exprSet3[,-c(1:2)]

test <- expr[,1:20]

expr <- as.data.frame(t(expr))

expr <- as.matrix(expr)

save(expr, file = "expr.Rdata")

####

library(GSVA)

gsva_data <- gsva(expr,cellMarker, method = "ssgsea")

test <- gsva_data[1:10,1:10]

tcga_gsva <- as.data.frame(t(gsva_data))

test <- tcga_gsva[1:10,1:10]

save(tcga_gsva,file = "tcga_gsva.Rdata")

####

rm(list = ls())

load("exprSet1.Rdata")

load("tcga_gsva.Rdata")

dd <- data

test1 <- dd[,1:10]

library(dplyr)

library(tibble)

dd1 <- dd[,c(1:2)]

tcga_gsva1 <- tcga_gsva %>%

rownames_to_column("ID") %>%

inner_join((dd1 <- dd1 %>%

rownames_to_column("ID")),by="ID") %>%

dplyr::select("ID","riskScore","risk",everything()) %>%

column_to_rownames("ID")

table(tcga_gsva1$risk)

save(tcga_gsva1,file = "TCGA_ssGSEA.Rdata")

####

rm(list = ls())

load(file = "TCGA_ssGSEA.Rdata")

library(dplyr)

library(tidyr)

dd1 <- tcga_gsva1 %>%

pivot_longer(cols=3:30,

names_to= "celltype",

values_to = "NES")

dd1$celltype <- factor(dd1$celltype,levels = c("Eosinophil","Immature B cell","Mast cell","T follicular helper cell","Activated B cell",

"Plasmacytoid dendritic cell","Immature dendritic cell","Central memory CD4 T cell",

"MDSC","Activated CD8 T cell","Monocyte","Effector memeory CD8 T cell","Macrophage",

"Activated dendritic cell","Effector memeory CD4 T cell","Natural killer cell",

"Regulatory T cell","Type 1 T helper cell","Central memory CD8 T cell",

"Type 17 T helper cell","Gamma delta T cell","Natural killer T cell",

"CD56bright natural killer cell","CD56dim natural killer cell","Neutrophil",

"Activated CD4 T cell","Type 2 T helper cell","Memory B cell"),ordered = F)

library(ggplot2)

library(ggpubr)

ggplot(data =dd1, aes(x = celltype, y = NES))+

geom_boxplot(aes(fill = risk),position = position_dodge(1),width=.3,outlier.shape = NA)+

geom_violin(aes(colour = risk),position = position_dodge(1),scale = "width",fill=NA)+

theme_bw()+

theme(axis.text.x = element_text(angle = 45, hjust = 1,vjust = 1, colour = "black"))+

stat_compare_means(aes(group=risk), label = "p.signif")

library(export)

graph2ppt(file = "ssGSEA_1.txt",width=19,height=7)

####

rm(list = ls())

load(file = "TCGA_ssGSEA.Rdata")

exprSet1 <- as.data.frame(t(tcga_gsva1))

gene <- "riskScore"

####

batch_cor <- function(gene){

y <- as.numeric(exprSet1[gene,])

rownames <- rownames(exprSet1)[3:30]

do.call(rbind,future_lapply(rownames, function(x){

dd <- cor.test(as.numeric(exprSet1[x,]),y,method="spearman")

data.frame(gene=gene,mRNAs=x,cor=dd$estimate,p.value=dd$p.value )

}))

}

library(future.apply)

plan(multiprocess)

system.time(dd <- batch_cor(gene))

save(dd,file = "dd_cor.Rdata")

####

rm(list = ls())

library(ggpubr)

library(reshape)

load("dd_cor.Rdata")

write.table(dd,file="dd.xls",sep="\t",row.names=F,quote=F)

bc <- read.table("dd.txt",header = T,sep="\t",check.names=F)

bc$Cor <- abs(bc$cor)

library(tibble)

library(dplyr)

bc$mRNAs <- factor(bc$mRNAs,levels = c("Eosinophil","Immature B cell","Mast cell","T follicular helper cell",

"Activated B cell","Plasmacytoid dendritic cell","Immature dendritic cell",

"Central memory CD4 T cell","MDSC","Activated CD8 T cell","Monocyte",

"Effector memeory CD8 T cell","Macrophage","Activated dendritic cell",

"Effector memeory CD4 T cell","Natural killer cell","Regulatory T cell",

"Type 1 T helper cell","Central memory CD8 T cell","Type 17 T helper cell",

"Gamma delta T cell","Natural killer T cell","CD56bright natural killer cell",

"CD56dim natural killer cell","Neutrophil","Activated CD4 T cell",

"Type 2 T helper cell","Memory B cell"),ordered = F)

ggplot(bc, aes(mRNAs, cor),rotate = T) +

geom_segment(aes(xend=mRNAs, yend = 0),linetype = "dashed",colour = "orange") +

geom_point(aes(color = p.value, size = Cor)) +

scale_color_viridis_c(guide=guide_colorbar(reverse=TRUE)) +

scale_color_continuous(low="brown1", high="cyan", guide=guide_colorbar(reverse=TRUE))+

scale_size_continuous(range=c(2, 8)) +

theme_minimal() +

xlab("Cor") +

ylab(NULL)+

theme(axis.text.x = element_text(angle = 45, hjust = 1,vjust = 1, colour = "black"))

library(export)

graph2ppt(file = "bbt——3.ppt",width=12,height=5)

####

#### immune cell survival

####

rm(list = ls())

library(survival)

library(survminer)

load(file = "TCGA_ssGSEA.Rdata")

immune_cell_exp <- tcga_gsva1[,-1]

load("TCGA_LUAD_500tumor_OS.Rdata")

sur <- exprSet3[,c(1,2)]

library(tibble)

library(dplyr)

immune_cell_exp_1 <- immune_cell_exp %>%

rownames_to_column("id") %>%

inner_join((sur <- sur %>%

rownames_to_column("id")),by = "id") %>%

dplyr::select("id","futime","fustat","risk",everything())

save(immune_cell_exp_1,file = "immune_cell_exp_1.Rdata")

####

rm(list = ls())

load(file = "immune_cell_exp_1.Rdata")

coxdata <- immune_cell_exp_1

library(dplyr)

coxdata <- coxdata %>%

filter(futime != "NA")

test1 <- coxdata[,1:10]

coxdata$futime <- coxdata$futime/365

rt <- coxdata

test1 <- rt[,1:10]

genes <- colnames(rt)[5:32]

res2 <- data.frame()

for (i in 1:length(genes)) {

print(i)

surv =as.formula(paste('Surv(futime, fustat)~', "group"))

group = ifelse(rt[,genes[i]] > median(rt[,genes[i]]),"high","low")

if(length(table(group))==1) next

data = cbind(rt[,2:3],group)

x = survdiff(surv, data = data)

pValue=1-pchisq(x$chisq,df=1)

res2[i,1] = genes[i]

res2[i,2] = pValue

}

names(res2) <- c("ID","pValue_log")

####

rm(list = ls())

load(file = "TCGA_ssGSEA.Rdata")

####

library(tidyverse)

library(ggplot2)

library(ggpubr)

library(cowplot)

library(ggExtra)

####

#### "Activated_B_cell" "Immature_B_cell" "Immature_dendritic_cell"

#### "Natural_killer_cell" "Plasmacytoid_dendritic_cell" "Eosinophil"

####

p <- ggplot(tcga_gsva1,aes_string(x = "riskScore",y = "Eosinophil")) +

geom_point(size = 2,color = '#EC0101',alpha = 0.5) +

theme_bw() +

geom_smooth(method = 'lm',se = T,color = '#F9B208',size = 1.5,fill = '#FEA82F') +

stat_cor(method = "spearman",digits = 2,size=6)

####

ggplot(tcga_gsva1,aes_string(x = "riskScore",y = "Eosinophil")) +

geom_point(size = 2,color = '#EC0101',alpha = 0.5) +

theme_bw() +

theme(axis.title = element_text(size = 16),

axis.text = element_text(size = 14),

axis.ticks.length = unit(0.25,'cm'),

axis.ticks = element_line(size = 1),

panel.border = element_rect(size = 1.5),

panel.grid = element_blank()

) +

geom_smooth(method = 'lm',se = T,color = '#F9B208',size = 1.5,fill = '#FEA82F') +

stat_cor(method = "spearman",digits = 2,size=6)

ggMarginal(p,type = "densigram",

xparams = list(binwidth = 0.1, fill = "#B3E283",size = .7),

yparams = list(binwidth = 0.1, fill = "#8AB6D6",size = .7))

library(export)

graph2ppt(file = "Eosinophil.pptx",width=4,height=4)

####

rm(list = ls())

load(file = "TCGA_ssGSEA.Rdata")

sur <- read.table("train_risk.txt",header = T,sep="\t",check.names=F)

sur <- sur[,c(1:3)]

library(tibble)

library(dplyr)

tcga_gsva2 <- tcga_gsva1 %>%

rownames_to_column("id") %>%

inner_join(sur, by = "id") %>%

column_to_rownames("id") %>%

dplyr::select(-c("riskScore","risk")) %>%

dplyr::select("futime","fustat",everything())

library(survival)

library(survminer)

exprSet <- tcga_gsva2

####

#### "Activated B cell" "Immature B cell" "Immature dendritic cell"

#### "Natural killer cell" "Plasmacytoid dendritic cell"

####

gene <- "Eosinophil"

####

if(T){

rt <- exprSet[,c("futime","fustat",gene)]

colnames(rt)[3] <- c("riskScore")

rt$risk <- ifelse(rt$riskScore > median(rt$riskScore),"high","low")

table(rt$risk)

cutoff <- sort(rt$riskScore)[sum(rt$risk =="low")]

####

my.surv <- Surv(rt$futime, rt$fustat)

group <- rt$risk

survival_dat <- data.frame(group = group)

fit <- survfit(my.surv ~ group)

####

dd <- as.data.frame(summary(fit)$table)

median <- c(round(dd$median[1],3),round(dd$median[2],3))

####

if(T){

group <- factor(group, levels = c("low", "high"))

data.survdiff <- survdiff(my.surv ~ group)

p.val = 1 - pchisq(data.survdiff$chisq, length(data.survdiff$n) - 1)

x = summary(coxph(Surv(futime, fustat)~riskScore, data = rt))

rt <- rt[order(rt[,"riskScore"],decreasing = T),]

ggsurvplot(fit, data = survival_dat ,

ggtheme = theme_bw(),

conf.int = T,

conf.int.style = "ribbon",

censor = T,

surv.median.line = "hv",

break.time.by = 2,

risk.table=T,

risk.table.height=.25,

risk.table.title="Numble at risk",

palette = c("darkorange","deepskyblue"),

ncensor.plot = FALSE,

font.legend = 12,

pval = paste(paste("pvalue =", p.val), paste("Median OS = ",median[1]," vs ",median[2]),sep = "\n"))

}

}

library(export)

graph2ppt(file= paste0(gene,"_sur.ppt"),width=5,height=5)

####

#### Chemosensitivity

####

rm(list = ls())

library(readxl)

library(impute)

library(limma)

rt1 <- read_excel(path = "DTP_NCI60_ZSCORE.xlsx",skip = 7)

colnames(rt1) <- rt1[1,]

rt1 <- rt1[-1,-c(67:68)]

table(rt1$`FDA status`)

rt1 <- rt1[rt1$`FDA status` %in% c("FDA approved","Clinical trial"),]

rt <- rt1[,-c(1,3:6)]

write.table(rt, file = "drug.xls", sep = "\t", row.names = F, quote = F)

rt <- as.matrix(rt)

rownames(rt) <- rt[,1]

drug <- rt[,2:ncol(rt)]

dimnames <- list(rownames(drug),colnames(drug))

data <- matrix(as.numeric(as.matrix(drug)),nrow = nrow(drug),dimnames = dimnames)

data <- data[,-34]

mat <- impute.knn(data)

drug <- mat$data

drug <- avereps(drug)

save(drug,file = "drug.Rdata")

####

exprSet <- read_excel(path = "RNA__RNA_seq_composite_expression.xls",skip = 9)

colnames(exprSet) <- exprSet[1,]

exprSet <- exprSet[-1,-c(2:6)]

library(tibble)

library(dplyr)

exprSet <- exprSet %>%

column_to_rownames("Gene name d")

dim(exprSet)

exprSet[1:4,1:4]

exp <- exprSet[c("MMP14","IL7R","TLR2","PCDH7","LAMP3","CCL20","BTG2"),]

is.na(exp)

exp <- exp[,-34]

exp <- as.data.frame(t(exp))

write.table(exp, file = "drug_exp.xls", sep = "\t", row.names = T, quote = F)

save(exp, file = "drug_exp.Rdata")

####

rm(list = ls())

load("drug.Rdata")

exp <- read.table("drug_exp.txt",header = T,sep="\t",check.names=F,row.names = 1)

drug_exp <- as.data.frame(t(exp))

####

outTab <- data.frame()

for (Drug in row.names(drug)) {

x <- as.numeric(drug_exp[8,])

y <- as.numeric(drug[Drug,])

corT <- cor.test(x,y,method = "spearman")

cor <- corT$estimate

pvalue <- corT$p.value

if(pvalue < 0.05){

outVector <- cbind(rownames(drug_exp)[8],Drug,cor,pvalue)

outTab <- rbind(outTab,outVector)

}

}

save(outTab,file = "riskScore.Rdata")

####

rm(list = ls())

load("riskScore.Rdata")

load("drug.Rdata")

exp <- read.table("drug_exp.txt",header = T,sep="\t",check.names=F,row.names = 1)

exp <- as.data.frame(t(exp))

drug <- as.data.frame(drug)

library(ggplot2)

exprSet1 <- rbind(exp,drug)

exprSet1 <- as.data.frame(t(exprSet1))

gene_gene <- "riskScore"

gene <- "Trametinib"

exprSet <- exprSet1[,c(which(colnames(exprSet1) == gene_gene),which(colnames(exprSet1) == gene))]

exprSet[,1] <- as.numeric(exprSet[,1])

exprSet[,2] <- as.numeric(exprSet[,2])

ggcorplot <- function(a,b){

corr_eqn <- function(x,y,digits=8) {

test <- cor.test(x,y,method="spearman")

paste(paste0("n = ",length(x)),

paste0("r = ",round(test$estimate,digits),"(Spearman)"),

paste0("p.value= ",round(test$p.value,digits)),

sep = ", ")

}

plot_df <- exprSet[,c(a,b)]

names(plot_df) <- c("geneA","geneB")

ggplot(plot_df,aes(geneA,geneB))+

geom_point(col="#984ea3")+

geom_smooth(method=lm, se=T,na.rm=T, fullrange=T,size=1.5,col="red")+

geom_rug(col="blue")+

theme_minimal()+

xlab(paste0(a))+

ylab(paste0(b))+

labs(title = paste0(corr_eqn(plot_df$geneA,plot_df$geneB)))+

theme(plot.title = element_text(hjust = 0.5))

}

ggcorplot(gene_gene,gene)

library(export)

graph2ppt(file = paste0(gene_gene,gene,".ppt"),width=3,height=3)
